# Dynamics of radiative Williamson hybrid nanofluid with entropy generation: significance in solar aircraft

**DOI:** 10.1038/s41598-022-13086-4

**Published:** 2022-05-26

**Authors:** Syed M. Hussain

**Affiliations:** Department of Mathematics, Faculty of Science, Islamic University of Madinah, Medina, 42351 Saudi Arabia

**Keywords:** Applied mathematics, Computational science

## Abstract

Sun based energy is the chief source of heat from the sun, and it utilizes in photovoltaic cells, sun-based power plates, photovoltaic lights and sun-based hybrid nanofluids. Specialists are currently exploring the utilization of nanotechnology and sun-based radiation to further develop flight effectiveness. In this analysis, a hybrid nanofluid is moving over an expandable sheet. Analysts are presently exploring the utilization of nanotechnology and sunlight-based radiation to further develop avionics productivity. To explore the heat transfer rate phenomenon, a hybrid nanofluid stream is moving towards a trough having a parabolic type shape and is located inside of solar airplane wings. The expression used to depict the heat transfer phenomenon was sun based thermal radiation. Heat transfer proficiency of airplane wings is evaluated with the inclusion of distinguished effects like viscous dissipation, slanted magnetic field and solar-based thermal radiations. The Williamson hybrid nanofluid past an expandable sheet was read up for entropy generation. The energy and momentum expressions were solved numerically with the utilization of the Keller box approach. The nano solid particles, which are comprised of copper (Cu) and Graphene oxide, are dispersed utilizing SA (Sodium alginate) as an ordinary liquid (GO). A huge number of control factors, for example, temperature, shear stress, velocity, frictional element along with Nusselt number are investigated in detail. Intensification of thermal conduction, viscous dissipation and radiation improve the performance of airplane wings subjected to heat transmission. Hybrid nanofluid performance is much better than the ordinary nanofluid when it comes to heat transmission analysis.

## Introduction

Generating and utilizing energy is the major source of pollution CO_2_ emissions, and because pollution and atmospheric CO_2_ concentrations are a hazard to life on our planet, reducing CO_2_ emissions and switching to carbon-free energy sources is a question of survival. The only renewable, as well as an eco-friendly source of energy, is solar energy having technological and distillate potentials beating our energy demands even in the future, according to forecasts of the Intergovernmental Panel on Climate Change (IPCC)^1^. Photovoltaics as well as considering solar-thermal power are pair of methods for assembling solar energy which is getting cheaper and more efficient as a consequence of recent technological advancements^2^. Solar energy uses are expanding, and we currently find it in heating, power generation, water treatment, lighting, transportation and ventilation^3–5^.

**Figure 1:**
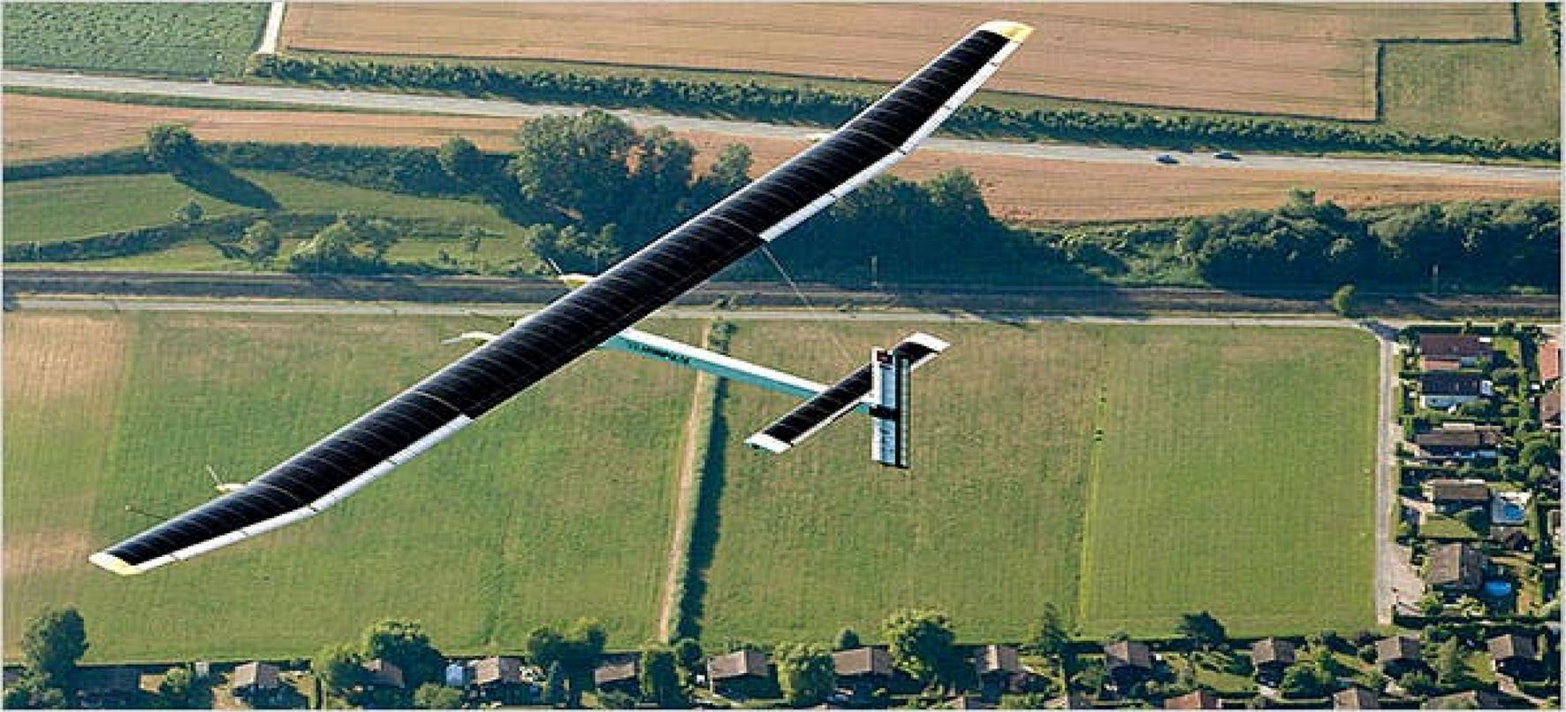
Solar aircraft.

The second most polluting industry which accounts for 27% of gass emissions of greenhouse regarding the total world is transportation^6,7^. Several triumphs to harness solar energy in boats, airplanes and vehicles have been made in recent years. Roland Boucher conceived as well as made the first-ever solar-powered auto aircraft, AstroFlight Sunrise, in November 1973. The first manned solar airplane, the Mauro Sun Riser, went to the skies six years later. These two solar planes were the first proof of concept for harnessing solar energy into powered aircraft. Piccard and Borschberg tried to fly Solar Impulse 2, which is a solar-powered aircraft around the world for the first time on 26 July 2016. They set 19 official aviation records including the longest-ever flight of 118 h for manned solar aircraft. Such events brought attention towards solar-powered aircraft while their great potentiality was proved regarding long-resolution, wide application prospects and pollution-free flight. Scientists started to work on the development as well as enhancement of such aircraft. Figure 1 represents the solar aircraft^8–10^.

Mingjian Wu led the researchers' team to discuss a series of researches about effect regarding flight conditions, Z-shaped wing^11^, solar cell efficiency, wingtip connection^12^, as ${\Lambda }$-shaped rotatable wing^13^ over flight resolution along with energy capability related to solar aircraft. Wang et al.^14^ utilized a flight optimization scheme for enhancement of flight performance as well as its endurance in solar aircraft with the implementation of the Gauss pseudo-spectral method.

Few suggestions were made about major changes into ordinary strategies for optimality achievement of long-endurance regarding solar-powered aircraft. Strategies include an adaptation of controlling changes about attitude angle plus the advantage of gravitational potential energy. Management planning regarding energy was proposed by Gao et al.^15^ for the extension of altitude potential and flight time regarding solar-powered aircraft. An auto solar-powered aircraft was mapped by Barbosa et al.^16^ that utilizes the storage system of hydrogen energy. They also introduced some tools and suggestions for avoiding the under-sizing as well as oversizing generation of power along with systems regarding storage.

Because solar energy is not available at night, solar aircraft operate on the cycle of day and night. During the day, they collect solar energy, use some of it, and store the rest for use at night. For performance improvement of such aircraft, the harvesting rate of solar energy should be improved in the daytime. Gao et al.^17^ studied the most up-to-date ways for collecting and storing the energy required for solar aircraft. The utilization of nanofluids as base fluids is a novel way to the improvement of solar collectors. Choi and Eastman^18^ said nanofluids are with extraordinary properties that are ideal regarding applications of heat transfer^19–23^. Subramani et al.^24^ reported that using Al_2_O_3_ nanofluid at 0.05% volume fraction helped in enhancing the efficiency of the collector by 3%. Rubbi et al.^25^ examined enhancement in efficiency regarding hybrid PV/T solar collector with the utilization of rare nanofluid that consists of soybean oil as working fluid along with MXene (Ti_3_C_2_) particles. Best efficiency was obtained for nanofluids based on soybean oil than ordinary base fluids. Abdelrazik et al.^26^ conducted a practical investigation about the influence of using optical filter based on nanofluids to improve the efficiency of a hybrid PV/thermal system. Reader may check^27,28^ for further interests.

Changes in volume %, nanoparticles size, temperature, and a few other factors can alter the thermal conductivity of nanofluids^29,30^. Chemicals and metallurgical mechanization, transport, power obstetrics, macroscopic products, and cancer medicine are among the industries^31,32^. As nanofluids are expensive for selection, hybrid nanofluids are more suitable for their const effectiveness and can be employed for the attainment of thermophysical factors^33^. Optimization of thermal behavior about automatic radiators was done by Abbas et al.^34^ by employing TiO_2_/Fe_2_O_3–_water hybrid nanofluid. Improvement in the rate of heat transfer was observed as 26.7%. Excess in volumetric fraction of nanoparticles by 0.009 vol% resulted in clogging that caused the stability of hybrid nanofluids to diminish as well as deterioration in radiator performance. Shoaib et al.^35^ presented 3-D MHD flow of hybrid nanofluid close to a rotational disk in the existence of thermal radiation, viscous dissipation as well as ohmic heating. Tong et al.^36^ discussed the impact of adding MWCNTs to a Fe_3_O_4_ nanofluid. They created hybrid nanofluid on conversion rates of photo-thermal, optical as well as thermal energy of nanofluid. MWCNTs contributed to enhancing heat transfer ability along with solar absorption. Thermal conductivity of ternary hybrid nanofluid with the incorporation of MWCNTs, titania and zinc oxide nanoparticles was estimated by Boroomandpour et al.^37^. The recent additions deal with the traditional nanofluids representing the heat and mass transmission considering the variety of physical circumstances reported by^38–61^.

An improvement in thermal conductivity of nanofluids was induced by an increment in their volume fraction. Meanwhile, sedimentation and clogging would be obtained if there is high concentration of nanoparticles. A solar collector of flat plate form was utilized by Hussein et al.^62^ to assess employment regarding covalent MWCNTs that were functionalized. According to Ashrae standard, FPSC has become more thermal effective by 85% than the use of mono nanofluid. Influence regarding the shape of nanoparticles on hybrid as well as mono nanofluids having MHD and convective flow was examined by Iftikhar et al.^63^. Jin et al.^64^ studied the solar energy assimilation efficiency of hybrid nanofluid while applying the direct absorption method. According to results, hybrid nanofluid Improvement was made in a system of photothermal conversion efficiency, addedly to the existence of an optimal mixture regarding the concentration of nanoparticles of hybrid nanofluid. Yıldırım and Yurddaş^65^ examined the performance of heat transfer regarding solar collector of U-tube form with the employment of hybrid nanofluid (SiO_2–_Cu), then made a comparison of it with Cu/water nanofluid performance. According to the results, the addition of SiO_2_ nanoparticles into Cu/water nanofluid resulted in improving the heat transfer ability of later along with eliminating the problem of precipitation. Yan et al.^66^ employed a non-Newtonian and two-phase model to study the effect of U-shaped tube absorber regarding thermal and hydraulic performance about parabolic solar collectors working with two fluids including hybrid nanofluid. Further, an innovative study representing the heat transmission features of hybrid nanofluids considering various physical situations has been inspected by^67–74^.

According to a previous literature survey, a few attempts has been made for analysis of entropy generation using hybrid nanofluids in solar systems. The performance of thermal systems is evaluated through analysis of entropy production. Armaghani et al.^75^ analyzed entropy generation of MHD and mixed convection Al_2_O_3–_Cu/water hybrid nanofluid flow in the L-shaped enclosure. The same hybrid nanofluid was utilized by Kashyap et al.^76^ for analysis of entropy generation as well as the effect of three different boundary constraints with a two-phase model. Moreover, artificial neural networks were employed by Khosravi et al.^77^ for the prediction of entropy generation about hybrid nanofluid which is flowing in microchannel fluid block. Optimization of entropy production, as well as heat transference of non-Newtonian hybrid nanofluid flow, was done by Shahsavar et al.^78^ in a concentric annulus. A flattened tube was exploited by Huminic et al.^79^ for entropy production analysis considering two-hybrid nanofluids. There is a greater performance of heat transference of MWCNT and Fe_3_O_4_ driven water hybrid nanofluid as compared with ND + Fe_3_O_4_/water. However, heat transfer was increased and production of system entropy was reduced when it comes to the base fluid. Similar explorations of entropy generation on the nanofluid with stretched sheet assuming various geometries is administered in^80–84^.

**Figure 2:**
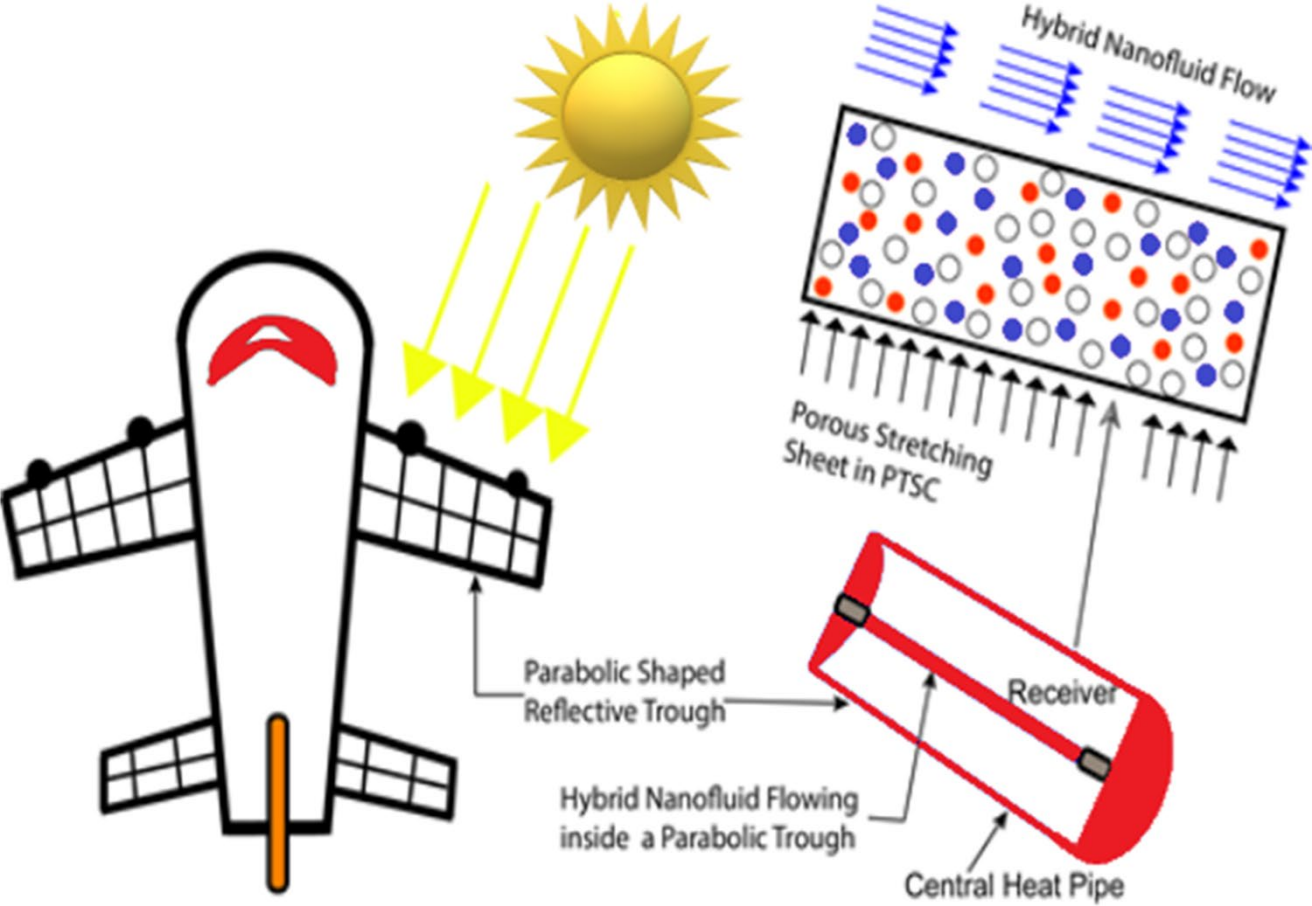
Systematic discription regarding current theoretical test.

The designation of Fig. 2 is such as to study the effectiveness of solar energy-based aircraft wings.

The trough has a parabolic shape called (PTSC) placed inside solar aircraft wings and collects solar thermal energy in the form of solar radiative scattering. Heat transmission on solar aircraft is investigated using hybrid nanofluids and analytical expressions of mathematics in the supplied study, which is said to be the first-ever endeavor. Aviation research will have a significant impact on the hunt for economically costly and alternative fuel supplies. By substituting standard nanofluids with the well-established hybrid nanofluids, the heat transfer rate amplifies. The work's results will be useful for new researchers because they were acquired using all cutting-edge material conditions.

The offered study can cover the gap lying within heat transfer using changing thermal conductivity and temperature as well as radiative Williamson hybrid nanofluid flow on a penetrating stretchy surface. Tiwari and Das model for nanofluids is employed to represent the mathematical flow of the nanofluid. In this work, hybrid nanoparticles of Graphene oxide (GO) together with copper (Cu) are employed as hybrid nanoparticles, with sodium alginate (SA) as the base fluid. The flow influence will be measured using entropy generation analysis. Hybrid nanoparticles are used in the research. The governing modelled equation of the current experimental model (Williamson hybrid nanofluid) will be converted into ordinary differential equations employing suitable similarity variables. The well-established and reliable numerical method labelled as Keller box method will be adopted to handle the system resultant ODEs utilizing the most relevant values of governing distinguished parameters. To better illustrate the numerical outcomes, graphs will be shown. The effects of solar-based thermal radiations, slippage effect at the surface of the sheet due to convection phenomenon, and slippage effect of the moving fluid will be thoroughly investigated.

### Aims regarding proposed model

In light of the current investigation, adopting the flow of hybrid nanofluid across a PTSC improves aircraft performance. Many PTSCs are positioned throughout the aircraft. The following are the reasons for this investigation: In the suggested paradigm, PVC cell sheets are substituted with PTSC. Because PTSC is a type of cylindrical form having a bigger surface area than typical PVC sheets, having more ability to collect and store solar energy. Solar aircraft may be manufactured and maintained at a minimal cost, making them economically viable. Scholars are striving to depend on solar-based thermal energy, notably in the sector of airplane manufacturing, as the price of oil constantly rises. According to the results of the experiment, introducing hybrid nanoparticles within the fluid moving across a PTSC improves heat transmission and delivers enormous energy. Thermal conduction and radiation, as well as viscous dissipation processes, are also present. Solar aircraft is environmentally friendly in comparison to other aircraft and does not pollute the atmosphere in any way.

## Formulations regarding flow model

The moving horizontal plate with irregular expanding velocity and isolated surface temperature are characterised as follows in the mathematical flow equations^85^:

1 $$ U_{w} \left( {x,t} \right) = \frac{bx}{{1 - \xi t}},\quad \yen_{w} \left( {x,t} \right) = \yen_{\infty } + \frac{{b^{*} x}}{1 - \xi t} $$

where $b$ and $b^{*}$ are original expanding rate and temperature variation, recpectively. ${\yen}_{w}$ and ${\yen}_{\infty }$ represent the temperature of surface and surrounds respectively. The plate is supposed to be slippery and temperature change is imperiled to the surface. Further, the hybrid nanofluid is expressed at 1^st^ with the addition of Copper (Cu) solid nano-particles in SA-based liquid at a interaction volume fraction ($\phi_{\beta }$) and its value was fixed at 0.09 while testing. Graphene oxide GO nanomolecules have been improved in mixture to get hybrid nanofluid having concentrated size ($\phi_{\lambda }$).

### Suppositions and terms of model

Following are the principals as well as conditions applicable to the flow model:

- 2-D flow having laminar and time-dependent features.
- Boundary-layer approximations.
- Tiwari-Das (Single phase) technique.
- Non-Newtonian WHNF.
- Penetrating medium.
- Flow with thermal radiative features.
- Flow having viscid dissipation properties.

The tensor of stress in Williamson type is specified as^86^

2 $$ S^{*} = - pI + \tau_{ij} , $$

where

3 $$ \tau_{ij} = \left[ {\mu_{\infty } + \frac{{\left( {\mu_{0} - \mu_{\infty } } \right)}}{{\left( {1 - \zeta \tilde{\gamma }} \right)}}} \right]A_{\beta } , $$

where $\tau_{ij}$, $\mu_{0}$, $\mu_{\infty }$, $\zeta > 0$ and $A_{\beta }$ represent the additional stress-tensor, limited viscidness when shear rate is zero, limited viscidness when shear rate is infinite and the 1st Rivlin–Erickson tensor respectively. $\tilde{\gamma }$ is:

4 $$ \tilde{\gamma } = \sqrt {\frac{1}{2}\pi } , $$

5 $$ \pi = trace\left( {A_{\beta }^{2} } \right). $$

Herein, we presumed $\mu_{\infty } = 0$ and $\tilde{\gamma } < 1$. Thus formula (3) can be inscribed as

6 $$ \tau_{ij} = \left[ {\frac{{\mu_{0} }}{{\left( {1 - \zeta \tilde{\gamma }} \right)}}} \right]A_{\beta } , $$

Which can discribed as follows by used binomial-expansion

7 $$ \tau_{ij} = \left[ {\mu_{0} \left( {1 + \zeta \tilde{\gamma }} \right)} \right]A_{\beta } . $$

**Figure 3:**
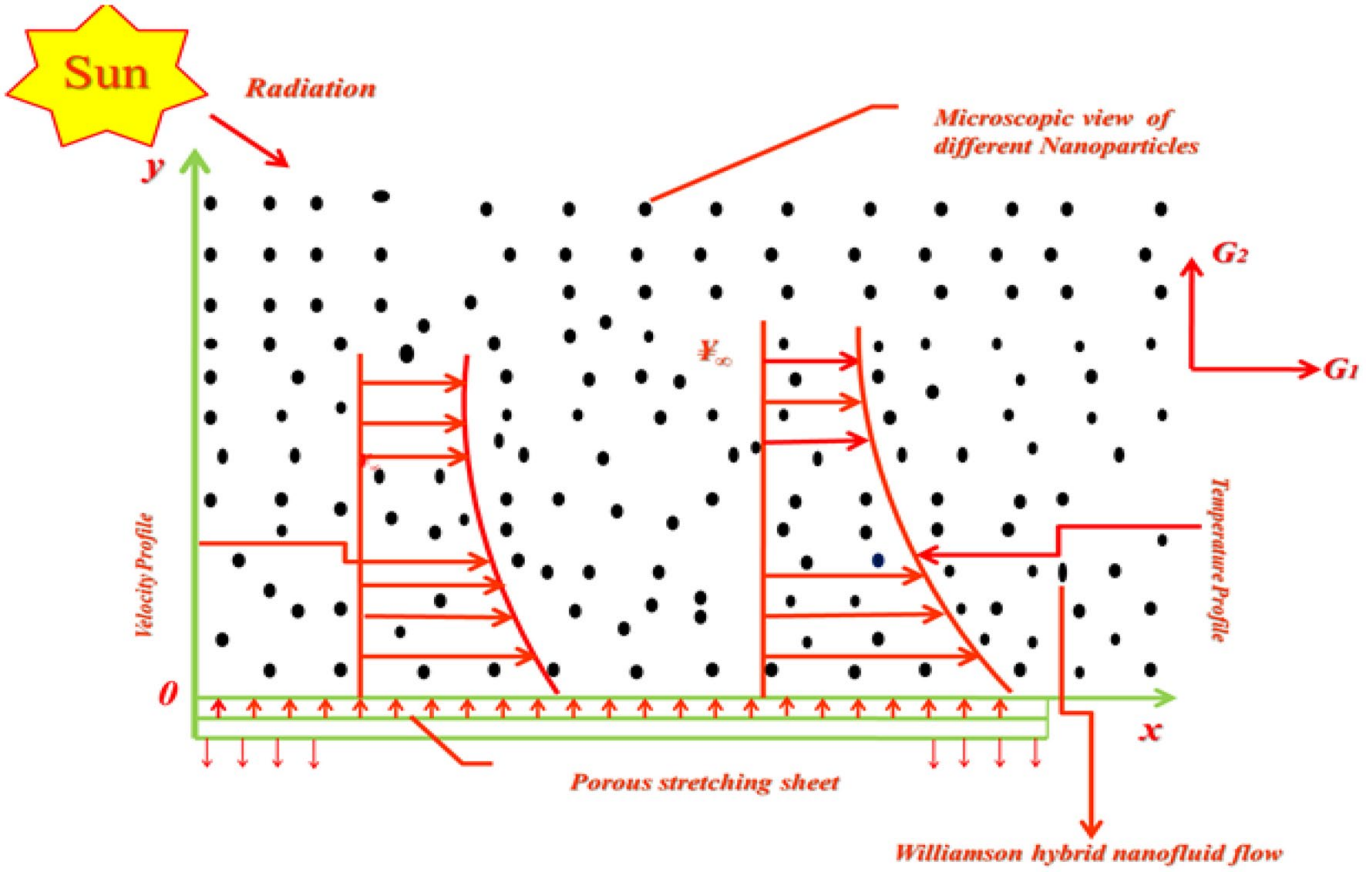
Flow model discription.

Inside PTSC, geometry of flow model is presented in Fig. 3 as:

The governing flow model equations^87^ regarding viscid WHNF under the aforementioned assumptions are given as

8 $$ \frac{{\partial G_{1} }}{\partial x} + \frac{{\partial G_{2} }}{\partial y} = 0, $$

9 $$ \frac{{\partial G_{1} }}{\partial t} + G_{1} \frac{{\partial G_{1} }}{\partial x} + G_{2} \frac{{\partial G_{1} }}{\partial y} = \frac{{\mu_{hnf} }}{{\rho_{hnf} }}\left( {\frac{{\partial^{2} G_{1} }}{{\partial y^{2} }}} \right) + \sqrt 2 \zeta \frac{{\mu_{hnf} }}{{\rho_{hnf} }}\left[ {\left( {\frac{{\partial G_{1} }}{\partial y}} \right)\left( {\frac{{\partial^{2} G_{1} }}{{\partial y^{2} }}} \right)} \right] - \frac{{\mu_{hnf} }}{{\rho_{hnf} k}}G_{1} , $$

10 $$ \frac{{\partial {\yen}}}{\partial t} + G_{1} \frac{{\partial {\yen}}}{\partial x} + G_{2} \frac{{\partial {\yen}}}{\partial y} = \frac{{k_{hnf} }}{{(\rho C_{p} )_{hnf} }}\left( {\frac{{\partial^{2} {\yen}}}{{\partial y^{2} }}} \right) - \frac{1}{{\left( {\rho C_{p} } \right)_{hnf} }}\left( {\frac{{\partial q_{r} }}{\partial y}} \right) + \frac{{\mu_{hnf} }}{{\left( {\rho C_{p} } \right)_{hnf} }}\left( {\frac{{\partial G_{1} }}{\partial y}} \right)^{2} . $$

Jamshed et al.^88^ reported the associated boundary constraints:

11 $$ G_{1} \left( {x,0} \right) = U_{w} + N_{w} \left( {1 + \zeta \left( {\frac{{\partial G_{1} }}{\partial y}} \right)\frac{{\partial G_{1} }}{\partial y}} \right),\quad G_{2} \left( {x,0} \right) = V_{w} , \quad - k_{0} \left( {\frac{{\partial {\yen}}}{\partial y}} \right) = h_{f} \left( {{\yen}_{w} - {\yen}} \right) $$

12 $$ G_{1} \to 0, \quad {\yen} \to {\yen}_{\infty } \;as\;y \to \infty . $$

Fluid velocity in vector form is well-defined as $\mathop{G}\limits^{\leftarrow}  = \left[ {G_{1} \left( {x,y,t} \right),G_{2} \left( {x,y,t} \right),0} \right]$. Time is denoted by $t$, ${\yen}$ represent temperature of the fluid. Here $N_{w}$ signifies the slip length. $V_{w}$ is representing the porosity of the encompassing plate while $k_{0}$ indicates the porousness of material.

The expressions of Table 1 summarize WNF variables of the material^89^.

**Table 1:** Thermo-physical structures related to nanofluids.

Characteristics	Nanoliquid
Dynamic viscosity $\left( \mu \right)$	$\mu_{nf} = \mu_{f} (1 - \phi )^{ - 2.5}$
Density $\left( \rho \right)$	$\rho_{nf} = \left( {1 - \phi } \right)\rho_{f} - \phi \rho_{s}$
Heat capacity $\left( {\rho C_{p} } \right)$	$(\rho C_{p} )_{nf} = \left( {1 - \phi } \right)(\rho C_{p} )_{f} - \phi (\rho C_{p} )_{s}$
Thermal conductivity $\left( \kappa \right)$	$\frac{{\kappa_{nf} }}{{\kappa_{f} }} = \left[ {\frac{{\left( {\kappa_{s} + 2\kappa_{f} } \right) - 2\phi \left( {\kappa_{f} - \kappa_{s} } \right)}}{{\left( {\kappa_{s} + 2\kappa_{f} } \right) + \phi \left( {\kappa_{f} - \kappa_{s} } \right)}}} \right]$

$\phi$ is representing size coefficien of nano solid-particle. $\mu_{f}$, $\rho_{f}$, $(C_{p} )_{f}$ and $\kappa_{f}$ are dynamic viscidness, intensity, operational heat capacitance as well as thermal conductance of standard fluid correspondingly. Extra factors $\rho_{s}$, $(C_{p} )_{s}$ and $\kappa_{s}$ are intensity, effective heat capacity and thermal conductivity of nanomolecules, respectively.

Content of WHNF variants is described in Table 2^90,91^.

**Table 2:** Thermo-physical features regarding hybrid nanofluids.

Features	Hybrid nanofluid
Viscosity $\left( \mu \right)$	$\mu_{hnf} = \mu_{f} (1 - \phi_{\beta } )^{ - 2.5} (1 - \phi_{\lambda } )^{ - 2.5}$
Density $\left( \rho \right)$	$\rho_{hnf} = \left[ {\left( {1 - \phi_{\lambda } } \right)\left\{ {\left( {1 - \phi_{\beta } } \right)\rho_{f} + \phi_{\beta } \rho_{{p_{1} }} } \right\}} \right] + \phi_{H} \rho_{{p_{2} }}$
Heat capacity $\left( {\rho C_{p} } \right)$	$(\rho C_{p} )_{hnf} = [\left( {1 - \phi_{\lambda } } \right)\{ \left( {1 - \phi_{\beta } } \right)(\rho C_{p} )_{f} + \phi_{\beta } (\rho C_{p} )_{{p_{1} }} \} ] + \phi_{\lambda } (\rho C_{p} )_{{p_{2} }}$
Thermal conductivity $\left( \kappa \right)$	$$\begin{aligned} \frac{{\kappa_{hnf} }}{{\kappa_{gf} }} & = \left[ {\frac{{\left( {\kappa_{{p_{2} }} + 2\kappa_{gf} } \right) - 2\phi_{\lambda } \left( {\kappa_{gf} - \kappa_{{p_{2} }} } \right)}}{{\left( {\kappa_{{p_{2} }} + 2\kappa_{gf} } \right) + \phi_{\lambda } \left( {\kappa_{gf} - \kappa_{{p_{2} }} } \right)}}} \right]; \\ \frac{{\kappa_{gf} }}{{\kappa_{f} }} & = \left[ {\frac{{\left( {\kappa_{{p_{1} }} + 2\kappa_{f} } \right) - 2\phi_{\beta } \left( {\kappa_{f} - \kappa_{{p_{1} }} } \right)}}{{\left( {\kappa_{{p_{1} }} + 2\kappa_{f} } \right) + \phi_{\beta } \left( {\kappa_{f} - \kappa_{{p_{1} }} } \right)}}} \right] \\ \end{aligned}$$

In Table 2, $\mu_{hnf}$, $\rho_{hnf}$, $\rho (C_{p} )_{hnf}$ and $\kappa_{hnf}$ are dynamical viscidness of hybrid nanofluid, intensity, specific thermal capacity and heat conductance. $\phi$ symbolizes volumetric coefficient of solid nanomolecules regarding mono nanofluid. $\phi_{hnf} = \phi_{\beta } + \phi_{\lambda }$ is the coefficient of nano-sized solid-particles for the mixed nanofluid. $\mu_{f}$, $\rho_{f}$, $(C_{p} )_{f}$, $\kappa_{f}$ plus $\sigma_{f}$ are representing dynamic viscidness, density, specific heat capacitance and thermal conductance regarding basefluid. $\rho_{{p_{1} }}$, $\rho_{{p_{2} }}$, $(C_{p} )_{{p_{1} }}$,$(C_{p} )_{{p_{2} }}$, $\kappa_{{p_{1} }}$ and $\kappa_{{p_{2} }}$ are respectively the intensities, specific heat capacitances as well as thermal conductances regarding nanomolecules.

The thermo-physical material features of sodium alginate, copper and graphene oxide used in the numerical computation during research study have been reported in Table 3^92,93^.

**Table 3:** Thermo-physical characteristics.

Thermo-physical	$\rho \left( {{\text{kg/m}}^{3} } \right)$	$c_{p} \left( {{\text{J/kg}}\;{\text{K}}} \right)$	$k\left( {\text{W/mK}} \right)$
Copper $\left( {{\text{Cu}}} \right)$	8933	385.0	401.00
Sodium alginate (SA)	989	4175	0.6376
Graphene oxide $\left( {{\text{GO}}} \right)$	1800	717	5000

Ensuing the research study of Brewster^94^, for an optically thick nanofluid, the radiation heat flux $q_{r}$ is written employing Rosseland approximation as:

13 $$ q_{r} = - \frac{{4\sigma^{*} }}{{3k^{*} }}\frac{{\partial {\yen}^{4} }}{\partial y}, $$

where $\sigma^{*}$ designates the Stefan-Boltzmann constant and $k^{*}$ shows the absorption coefficient.

## Dimensionless formulations model

To examine the solution of constitutive system (8)–(10) with the alligned boundary conditions (11)–(12), the stream function $\psi$ is defined as

14 $$ G_{1} = \frac{\partial \psi }{{\partial y}}, G_{2} = - \frac{\partial \psi }{{\partial x}}. $$

The specified similarity quantities are

15 $$ \chi \left( {x,y} \right) = \sqrt {\frac{b}{{\nu_{f} \left( {1 - \xi t} \right)}}} y,\;\;\psi \left( {x,y} \right) = \sqrt {\frac{{\nu_{f} b}}{{\left( {1 - \xi t} \right)}}} xf\left( \chi \right),\;\;\theta \left( \chi \right) = \frac{{{\yen} - {\yen}_{\infty } }}{{{\yen}_{w} - {\yen}_{\infty } }}. $$

into Eqs. (8)–(10). We get

16 $$ f^{\prime\prime\prime} + \phi_{a} \phi_{b} \left[ {ff^{\prime\prime} - f^{\prime 2} - A\left( {\frac{\chi }{2}f^{\prime\prime} + f^{\prime}} \right)} \right] + \lambda \left( {f^{\prime\prime}\;f^{\prime\prime\prime}} \right) - Kf^{\prime} = 0, $$

17 $$ \theta^{\prime\prime}\left( {1 + \frac{1}{{\phi_{d} }}P_{r} N_{r} } \right) + P_{r} \frac{{\phi_{c} }}{{\phi_{d} }}\left[ {f\theta^{\prime} - f^{\prime}\theta - A\left( {\theta + \frac{\chi }{2}\theta^{\prime}} \right) + \frac{{E_{c} }}{{\phi_{a} \phi_{c} }}f^{\prime \prime 2} } \right] = 0, $$

with

18 $$ \left. {\begin{array}{*{20}l} {f\left( 0 \right) = S,\;\;f^{\prime}\left( 0 \right) = 1 + {\Lambda }\left( {f^{\prime\prime}\left( 0 \right) + \frac{\lambda }{2}\left( {f^{\prime\prime}\left( 0 \right)} \right)^{2} } \right),\;\;\theta^{\prime}\left( 0 \right) = - B_{i} \left( {1 - \theta \left( 0 \right)} \right)} \hfill \\ {f^{\prime}\left( \chi \right) \to 0,\;\;\theta \left( \chi \right) \to 0,\;\;as\;\chi \to \infty } \hfill \\ \end{array} } \right\} $$

here $\phi^{\prime}_{i} s$ are $a \le i \le d$ in Eqs. (16)–(17) specify consecutive thermo-physical discriptions for WNH

19 $$ \phi_{a} = (1 - \phi_{1} )^{2.5} (1 - \phi_{2} )^{2.5} , \phi_{b} = \left( {1 - \phi_{2} } \right)\left[ {\left( {1 - \phi_{1} } \right) + \phi_{1} \frac{{\rho_{{p_{1} }} }}{{\rho_{f} }}} \right] + \phi_{2} \frac{{\rho_{{p_{2} }} }}{{\rho_{f} }}, $$

20 $$ \phi_{c} = \left( {1 - \phi_{2} } \right)\left\{ {\left( {1 - \phi_{1} } \right) + \phi_{1} \frac{{(\rho C_{p} )_{{p_{1} }} }}{{(\rho C_{p} )_{f} }}} \right\} + \phi_{2} \frac{{(\rho C_{p} )_{{p_{2} }} }}{{(\rho C_{p} )_{f} }}, $$

21 $$ \phi_{d} = \left[ {\frac{{\left( {\kappa_{{p_{2} }} + 2\kappa_{nf} } \right) - 2\phi_{2} \left( {\kappa_{nf} - \kappa_{{p_{2} }} } \right)}}{{\left( {\kappa_{{p_{2} }} + 2\kappa_{nf} } \right) + \phi_{2} \left( {\kappa_{nf} - \kappa_{{p_{2} }} } \right)}}} \right]\left[ {\frac{{\left( {\kappa_{{p_{1} }} + 2\kappa_{f} } \right) + \phi_{1} \left( {\kappa_{f} - \kappa_{{p_{1} }} } \right)}}{{\left( {\kappa_{{p_{1} }} + 2\kappa_{f} } \right) - 2\phi_{1} \left( {\kappa_{f} - \kappa_{{p_{1} }} } \right)}}} \right]. $$

Equation (2) is accurate. The representation ′ is demonstrating derivatives with respect to $\chi$. The default values of emerged flow parameters alongwith their expressions are mentioned in Table 4.

**Table 4:** Clarification of entrenched control constraints.

Symboles	Name	Formule	Default value
$A$	Unsteadiness parameter	$A = \frac{\xi }{d}$	0.3
$\lambda$	Williamson parameter	$\lambda = \zeta U_{w} \sqrt {\frac{{2{\text{b}}}}{{\nu_{f} }}}$	0.1
$P_{r}$	Prandtl number	$P_{r} = \frac{{\nu_{f} }}{{\alpha_{f} }}$	6.5
$\phi$	Volume fraction	–	0.18
$K$	Porous medium parameter	$K = \frac{{\nu_{f} \left( {1 - \xi t} \right)}}{bk}$	0.1
$S$	Suction/injection parameter	$S = - V_{w} \sqrt {\frac{1 - \xi t}{{\nu_{f} { }b}}}$	0.1
$N_{r}$	Thermal radiation parameter	$N_{r} = \frac{16}{3}\frac{{\sigma^{*} {\yen}_{\infty }^{3} }}{{\kappa^{*} \nu_{f} (\rho C_{p} )_{f} }}$	0.3
$\Lambda$	Velocity slip	$\Lambda = \sqrt {\frac{b}{{\nu_{f} \left( {1 - \xi t} \right)}}} N_{w}$	0.3
$B_{i}$	Biot number	$B_{i} = \frac{{h_{f} }}{{k_{0} }}\sqrt {\frac{{\nu_{f} \left( {1 - \xi t} \right)}}{b}}$	0.2
$E_{c}$	Eckert number	$E_{c} = \frac{{U_{w}^{2} }}{{(C_{p} )_{f} \left( {T_{w} - T_{\infty } } \right)}}$	0.2
$B_{r}$	Brinkman number	$B_{r} = \frac{{\mu_{f} U_{w}^{2} }}{{k_{f} \left( {{\yen}_{w} - {\yen}_{\infty } } \right)}}$	5.0

Drag force $\left( {C_{f} } \right)$ along with local Nusselt number $\left( {Nu_{x} } \right)$ is indicating the potential awareness which is controlling flow and is provided in detail as^87^

22 $$ C_{f} = \frac{{\tau_{w} }}{{\rho_{f} U_{w}^{2} }},\quad Nu_{x} = \frac{{xq_{w} }}{{k_{f} \left( {{\yen}_{w} - {\yen}_{\infty } } \right)}}, $$

here $\tau_{w}$ and $q_{w}$ are signifying thermal flux which is

23 $$ \tau_{w} = \mu_{hnf} \left( {\frac{{\partial G_{1} }}{\partial y} + \frac{\zeta }{\sqrt 2 }\left( {\frac{{\partial G_{1} }}{\partial y}} \right)^{2} } \right)_{y = 0} ,\quad q_{w} = - k_{hnf} \left( {1 + \frac{16}{3}\frac{{\sigma^{*} {\yen}_{\infty }^{3} }}{{\kappa^{*} \nu_{f} (\rho C_{p} )_{f} }}} \right)\left( {\frac{{\partial {\yen}}}{\partial y}} \right)_{y = 0} $$

Following expressions are attained when dimensionless transformations (15) are employed

24 $$ C_{f} Re_{x}^{\frac{1}{2}} = \frac{{f^{\prime\prime}\left( 0 \right)}}{{\phi_{a} }}\left( {1 + \frac{\lambda }{2}f^{\prime\prime}\left( 0 \right)} \right),\quad Nu_{x} Re_{x}^{{ - \frac{1}{2}}} = - \frac{{k_{hnf} }}{{k_{f} }}\left( {1 + N_{r} } \right)\theta^{\prime}\left( 0 \right), $$

where $C_{f}$ embodies the drag force coefficient. $Re_{x} = \frac{{u_{w} x}}{{\nu_{f} }}$ is the local Reynold’s number according to the elongated velocity $u_{w} \left( x \right)$.

## Keller-box technique

**Figure 4:**
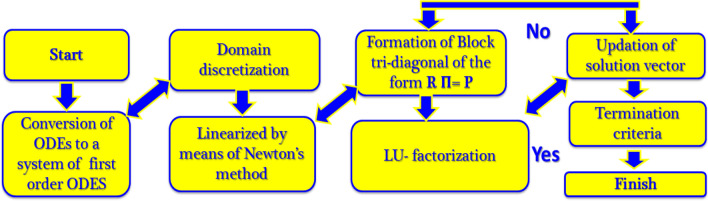
Chart of KBM steps.

As the convergence of the Keller-box method (KBM)^95^ can be obtained rapidly, solutions regarding governing equations of the model are obtained by employing it (Fig. 4). Localized solutions of (16)–(17) with constraints (18) can be gained with the help of KBM. The steps of KBM are specified as below:

### Stage 1: ODEs modification

At first, governed ODEs are moldified to 1st-order ODEs (16)–(18)

25 $$ z_{1} = f^{\prime}, $$

26 $$ z_{2} = z_{1}^{^{\prime}} , $$

27 $$ z_{3} = \theta^{\prime}, $$

28 $$ z_{2}^{^{\prime}} + \phi_{a} \phi_{b} \left[ {A\left( {z_{1} + \frac{\chi }{2}z_{2} } \right) - z_{1}^{2} + fz_{2} } \right] + \lambda \left( {z_{2} z_{3} } \right) - Kz_{1} = 0, $$

29 $$ z_{3}^{^{\prime}} \left( {1 + \frac{1}{{\phi_{d} }}P_{r} N_{r} } \right) + P_{r} \frac{{\phi_{c} }}{{\phi_{d} }}\left[ {fz_{3} - z_{1} \theta - A\left( {\theta + \frac{\chi }{2}z_{3} } \right) + \frac{{E_{c} }}{{\phi_{a} \phi_{c} }}z_{2}^{2} } \right] = 0, $$

30 $$ f\left( 0 \right) = S,z_{1} \left( 0 \right) = 1 + {\Lambda }z_{2} \left( 0 \right),z_{3} \left( 0 \right) = - B_{i} \left( {1 - \theta \left( 0 \right)} \right),z_{1} \left( \infty \right) \to 0,\theta \left( \infty \right) \to 0. $$

### Stage 2: domain discretization

**Figure 5:**
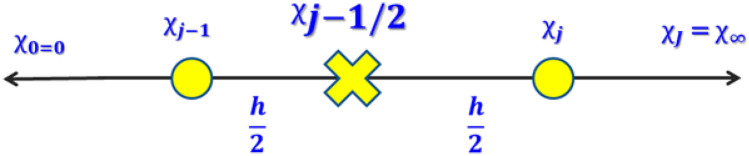
Rectangular grid of difference approximation.

The estimated solution can be computed when the domain procedure is discretized. Normally, discretization helps the field to divide into equal sizes of the grid (Fig. 5). High estimations result in less grid with the help of the computational outcomes.

$$ \chi_{0} = 0,\;\;\chi_{j} = \chi_{j - 1} + h,\;\;j = 1,2,3, \ldots ,J - 1,\;\;\chi_{J} = \chi_{\infty } . $$

The $j$ indicates the position of the coordinates that are used in $h$-spacing in horizontal direction. As there is no initial estimation, so obtained solution is unsertain. So $\Omega = 0$ and $\Omega = \infty$ are initial values to guess, as temperature variations, speed, temperatures and entropy outlines are important to find out. Estimated solutions are obtained when resulting outcomes are found, provided that they satisfy the boundary conditions regarding the problem. According to observation, when many initial estimations are being done, final results may seem to be equal. However, varied approximations are conducted when time and iterations are taken.

Centre difference formulae are employed to obtain differences formulas. Replacements are done for average functions. The 1st order ODEs (25)–(29) are converted into the next series of nonlinear algebraic expressions.

31 $$ \frac{{(z_{1} )_{j} + (z_{1} )_{j - 1} }}{2} = \frac{{f_{j} - f_{j - 1} }}{h}, $$

32 $$ \frac{{(z_{2} )_{j} + (z_{2} )_{j - 1} }}{2} = \frac{{(z_{1} )_{j} - (z_{1} )_{j - 1} }}{h}, $$

33 $$ \frac{{(z_{3} )_{j} + (z_{3} )_{j - 1} }}{2} = \frac{{\theta_{j} - \theta_{j - 1} }}{h}, $$

34 $$ \begin{aligned} & \left( {\frac{{(z_{2} )_{j} - (z_{2} )_{j - 1} }}{h}} \right) - \phi_{a} \phi_{b} \left[ {A\left\{ {\left( {\frac{{(z_{1} )_{j} + (z_{1} )_{j - 1} }}{2}} \right) + \frac{\chi }{2}\left( {\frac{{(z_{2} )_{j} + (z_{2} )_{j - 1} }}{2}} \right)} \right\}} \right] \\ & \quad - \phi_{a} \phi_{b} \left[ {\left( {\frac{{(z_{1} )_{j} + (z_{1} )_{j - 1} }}{2}} \right)^{2} - \left( {\frac{{f_{j} + f_{j - 1} }}{2}} \right)\left( {\frac{{(z_{2} )_{j} + (z_{2} )_{j - 1} }}{2}} \right)} \right] \\ & \quad + \left[ {\lambda \left( {\frac{{(z_{2} )_{j} + (z_{2} )_{j - 1} }}{2}} \right)\left( {\frac{{(z_{3} )_{j} + (z_{3} )_{j - 1} }}{2}} \right) - K\left( {\frac{{(z_{1} )_{j} + (z_{1} )_{j - 1} }}{2}} \right)} \right] \\ \end{aligned} $$

35 $$ \begin{aligned} & \left( {\frac{{(z_{3} )_{j} - (z_{3} )_{j - 1} }}{h}} \right)\left( {1 + \frac{1}{{\phi_{d} }}P_{r} N_{r} } \right) + P_{r} \frac{{\phi_{c} }}{{\phi_{d} }}\left[ {\left( {\frac{{f_{j} + f_{j - 1} }}{2}} \right)\left( {\frac{{(z_{3} )_{j} + (z_{3} )_{j - 1} }}{2}} \right)} \right] \\ & \quad + Pr\frac{{\phi_{c} }}{{\phi_{d} }}\left[ { - \left( {\frac{{z_{1j} + z_{1j - 1} }}{2}} \right)\left( {\frac{{\theta_{j} + \theta_{j - 1} }}{2}} \right) - A\left\{ {\left( {\frac{{\theta_{j} + \theta_{j - 1} }}{2}} \right) + \frac{\chi }{2}\left( {\frac{{(z_{3} )_{j} + (z_{3} )_{j - 1} }}{2}} \right)} \right\}} \right] \\ & \quad + P_{r} \frac{{\phi_{c} }}{{\phi_{d} }}\left[ {\frac{{E_{c} }}{{\phi_{a} \phi_{c} }}\left( {\frac{{z_{2j} + z_{2j - 1} }}{2}} \right)^{2} } \right] = 0. \\ \end{aligned} $$

### Stage 3: linearization of expressions employing Newton technique

Resultant equations are made linear when Newton technique is applied. $\left( {i + 1} \right){\text{th}}$ iteration obtained through previous formulas is:

36 $$ ()_{j}^{{\left( {i + 1} \right)}} = ()_{j}^{\left( i \right)} + {\Pi }()_{j}^{\left( i \right)} . $$

Following linear equation is obtained as we substituted the formula into Eqs. (31–35) and ignored the higher orders of $\Pi_{j}^{i}$ from 2 to above.

37 $$ \Pi f_{j} - \Pi f_{j - 1} - \frac{1}{2}h\left( {\Pi (z_{1} )_{j} + \Pi (z_{1} )_{j - 1} } \right) = (r_{1} )_{{j - \frac{1}{2}}} , $$

38 $$ \Pi (z_{1} )_{j} - \Pi (z_{1} )_{j - 1} - \frac{1}{2}h\left( {\Pi (z_{2} )_{j} + \Pi (z_{2} )_{j - 1} } \right) = (r_{2} )_{{j - \frac{1}{2}}} , $$

39 $$ \Pi \theta_{j} - \Pi \theta_{j - 1} - \frac{1}{2}h\left( {\Pi (z_{3} )_{j} + \Pi (z_{3} )_{j - 1} } \right) = (r_{3} )_{{j - \frac{1}{2}}} , $$

40 $$ \begin{aligned} & (a_{1} )_{j} \Pi f_{j} + (a_{2} )_{j} \Pi f_{j - 1} + (a_{3} )_{j} \Pi z_{1j} + (a_{4} )_{j} \Pi z_{1j - 1} + (a_{5} )_{j} \Pi z_{2j} + (a_{6} )_{j} \Pi z_{2j - 1} \\ & \quad + (a_{7} )_{j} \Pi \theta_{j} + (a_{8} )_{j} \Pi \theta_{j - 1} + (a_{9} )_{j} \Pi (z_{3} )_{j} + (a_{10} )_{j} \Pi (z_{3} )_{j - 1} = (r_{4} )_{{j - \frac{1}{2}}} , \\ \end{aligned} $$

41 $$ \begin{aligned} & (b_{1} )_{j} \Pi f_{j} + (b_{2} )_{j} \Pi f_{j - 1} + (b_{3} )_{j} \Pi z_{1j} + (b_{4} )_{j} \Pi z_{1j - 1} + (b_{5} )_{j} \Pi z_{2j} + (b_{6} )_{j} \Pi z_{2j - 1} \\ & \quad + (b_{7} )_{j} \Pi \theta_{j} + (b_{8} )_{j} \Pi \theta_{j - 1} + (b_{9} )_{j} \Pi (z_{3} )_{j} + (b_{10} )_{j} \Pi (z_{3} )_{j - 1} = (r_{5} )_{{j - \frac{1}{2}}} . \\ \end{aligned} $$

where

42 $$ (r_{1} )_{{j - \frac{1}{2}}} = - f_{j} + f_{j - 1} + \frac{h}{2}(z_{1} )_{j} + \left( {(z_{1} )_{j - 1} } \right), $$

43 $$ (r_{2} )_{{j - \frac{1}{2}}} = - (z_{1} )_{j} + (z_{1} )_{j - 1} + \frac{h}{2}\left( {(z_{2} )_{j} + (z_{2} )_{j - 1} } \right), $$

44 $$ (r_{3} )_{{j - \frac{1}{2}}} = - \theta_{j} + \theta_{j - 1} + \frac{h}{2}\left( {(z_{3} )_{j} + (z_{3} )_{j - 1} } \right), $$

45 $$ \begin{aligned} (r_{4} )_{{j - \frac{1}{2}}} & = - h\left[ { - \left( {\frac{{(z_{2} )_{j} - (z_{2} )_{j - 1} }}{h}} \right) + \left( {\phi_{a} \phi_{b} \left( {A\left( {\frac{{(z_{1} )_{j} + (z_{1} )_{j - 1} }}{2} + \chi \frac{{(z_{2} )_{j} - (z_{2} )_{j - 1} }}{4}} \right)} \right)} \right)} \right] \\ & \quad - h\left[ { - \phi_{a} \phi_{b} \left( {\left( {\frac{{(z_{1} )_{j} + (z_{1} )_{j - 1} }}{2}} \right)^{2} + \left( {\frac{{f_{j} + f_{j - 1} }}{2}} \right)\left( {\frac{{(z_{2} )_{j} + (z_{2} )_{j - 1} }}{2}} \right)} \right)} \right] + h\left[ {\lambda \left( {\left( {\frac{{(z_{2} )_{j} + (z_{2} )_{j - 1} }}{2}} \right)\left( {\frac{{(z_{3} )_{j} + (z_{3} )_{j - 1} }}{2}} \right)} \right) - K\left( {\frac{{(z_{1} )_{j} + (z_{1} )_{j - 1} }}{2}} \right)} \right], \\ \end{aligned} $$

46 $$ \begin{aligned} (r_{5} )_{{j - \frac{1}{2}}} & = - h\left[ {\left( {\frac{{((z_{3} )_{j} - \left( {z_{3} )_{j - 1} } \right)}}{h}} \right)\left( {1 + \frac{1}{{\phi_{d} }}P_{r} N_{r} } \right) + \frac{{\phi_{c} P_{r} }}{{\phi_{d} }}\left( {\frac{{\left( {f_{j} + f_{j - 1} } \right)((z_{3} )_{j} + \left( {z_{3} )_{j - 1} } \right)}}{4}} \right)} \right] \\ & \quad + h\frac{{\phi_{c} P_{r} }}{{\phi_{d} }}\left[ {\left( {\frac{{((z_{3} )_{j} + \left( {z_{3} )_{j - 1} } \right)\left( {z_{1j} + z_{1j - 1} } \right)}}{4}} \right)} \right] + h\frac{{\phi_{c} P_{r} }}{{\phi_{d} }}\left[ {A\left( {\frac{{\left( {\theta_{j} + \theta_{j - 1} } \right)}}{2} + \chi \frac{{((z_{3} )_{j} + \left( {z_{3} )_{j - 1} } \right)}}{4}} \right)\left( {\frac{{\theta_{j} + \theta_{j - 1} }}{2}} \right)} \right] \\ & \quad - h\frac{{\phi_{c} P_{r} }}{{\phi_{d} }}\left[ {\frac{{E_{c} }}{{\phi_{a} \phi_{c} }}\left( {\frac{{z_{2j} + z_{2j - 1} }}{2}} \right)^{2} } \right]. \\ \end{aligned} $$

The boundary conditions become

47 $$ \Pi f_{0} = 0,\;\Pi (z_{1} )_{0} = 0,\;\Pi (z_{3} )_{0} = 0,\;\Pi (z_{1} )_{J} = 0,\;\Pi \theta_{J} = 0. $$

The method is complete when boundary conditions are satisfied even for the whole set of iterations. So initial estimation is employed to obtain the actual values of every iteration.

### Stage 4: the block-tridiagonal array

A tridiagonal-block structure is utilized in linearized differential Eqs. (37–41). The method is as follows in a matrix–vector.

For $$j = 1;$$

48 $$ \Pi f_{1} - \Pi f_{0} - \frac{1}{2}h\left( {\Pi (z_{1} )_{1} + \Pi (z_{1} )_{0} } \right) = (r_{1} )_{{1 - \frac{1}{2}}} , $$

49 $$ \Pi (z_{1} )_{1} - \Pi (z_{1} )_{0} - \frac{1}{2}h\left( {\Pi (z_{2} )_{1} + \Pi (z_{2} )_{0} } \right) = (r_{2} )_{{1 - \frac{1}{2}}} , $$

50 $$ \Pi \theta_{1} - \Pi \theta_{0} - \frac{1}{2}h\left( {\Pi (z_{3} )_{1} + \Pi (z_{3} )_{0} } \right) = (r_{3} )_{{1 - \frac{1}{2}}} , $$

51 $$ \begin{aligned} & (a_{1} )_{1} \Pi f_{1} + (a_{2} )_{1} \Pi f_{0} + (a_{3} )_{1} \Pi z_{11} + (a_{4} )_{1} \Pi z_{10} + (a_{5} )_{1} \Pi z_{21} + (a_{6} )_{1} \Pi z_{20} \\ & \quad + (a_{7} )_{1} \Pi \theta_{j} + (a_{8} )_{1} \Pi \theta_{0} + (a_{9} )_{1} \Pi (z_{3} )_{1} + (a_{10} )_{1} \Pi (z_{3} )_{0} = (r_{4} )_{{1 - \frac{1}{2}}} , \\ \end{aligned} $$

52 $$ \begin{aligned} & (b_{1} )_{1} \Pi f_{1} + (b_{2} )_{1} \Pi f_{0} + (b_{3} )_{1} \Pi z_{11} + (b_{4} )_{1} \Pi z_{10} + (b_{5} )_{1} \Pi z_{21} + (b_{6} )_{1} \Pi z_{20} \\ & \quad + (b_{7} )_{1} \Pi \theta_{1} + (b_{8} )_{1} \Pi \theta_{0} + (b_{9} )_{1} \Pi (z_{3} )_{1} + (b_{10} )_{1} \Pi (z_{3} )_{0} = (r_{5} )_{{1 - \frac{1}{2}}} . \\ \end{aligned} $$

In array structure,

53 $$ \begin{aligned} & \left[ {\begin{array}{*{20}l} 0 \hfill & 0 \hfill & 1 \hfill & 0 \hfill & 0 \hfill \\ { - h/2} \hfill & 0 \hfill & 0 \hfill & { - h/2} \hfill & 0 \hfill \\ 0 \hfill & { - h/2} \hfill & 0 \hfill & 0 \hfill & { - h/2} \hfill \\ {(a_{2} )_{1} } \hfill & {(a_{10} )_{1} } \hfill & {(a_{3} )_{1} } \hfill & {(a_{1} )_{1} } \hfill & {(a_{9} )_{1} } \hfill \\ {(b_{2} )_{1} } \hfill & {(b_{10} )_{1} } \hfill & {(b_{3} )_{1} } \hfill & {(b_{1} )_{1} } \hfill & {(b_{9} )_{1} } \hfill \\ \end{array} } \right]\left[ {\begin{array}{*{20}l} {\Pi (z_{2} )_{0} } \hfill \\ {\Pi (\theta )_{0} } \hfill \\ {\Pi (f)_{1} } \hfill \\ {\Pi (z_{2} )_{1} } \hfill \\ {\Pi (z_{3} )_{1} } \hfill \\ \end{array} } \right] \\ & \quad + \left[ {\begin{array}{*{20}l} { - h/2} \hfill & 0 \hfill & 0 \hfill & 0 \hfill & 0 \hfill \\ 1 \hfill & 0 \hfill & 0 \hfill & 0 \hfill & 0 \hfill \\ 0 \hfill & 1 \hfill & 0 \hfill & 0 \hfill & 0 \hfill \\ {(a_{5} )_{1} } \hfill & {(a_{7} )_{1} } \hfill & 0 \hfill & 0 \hfill & 0 \hfill \\ {(b_{5} )_{1} } \hfill & {(b_{7} )_{1} } \hfill & 0 \hfill & 0 \hfill & 0 \hfill \\ \end{array} } \right]\left[ {\begin{array}{*{20}l} {\Pi (z_{1} )_{1} } \hfill \\ {\Pi (\theta )_{1} } \hfill \\ {\Pi (f)_{2} } \hfill \\ {\Pi (z_{2} )_{2} } \hfill \\ {\Pi (z_{3} )_{2} } \hfill \\ \end{array} } \right] = \left[ {\begin{array}{*{20}l} {(r_{1} )_{\frac{1}{2}} } \hfill \\ {(r_{2} )_{\frac{1}{2}} } \hfill \\ {(r_{3} )_{\frac{1}{2}} } \hfill \\ {(r_{4} )_{\frac{1}{2}} } \hfill \\ {(r_{5} )_{\frac{1}{2}} } \hfill \\ \end{array} } \right]. \\ \end{aligned} $$

That is

54 $$ \left[ {A_{1} } \right]\left[ {\Pi_{1} \left] + \right[C_{1} } \right]\left[ {\Pi_{2} \left] = \right[r_{1} } \right]. $$

For $$j = 2;$$

55 $$ \Pi f_{2} - \Pi f_{1} - \frac{1}{2}h\left( {\Pi (z_{1} )_{2} + \Pi (z_{1} )_{1} } \right) = (r_{1} )_{{1 - \frac{1}{2}}} , $$

56 $$ \Pi (z_{1} )_{2} - \Pi (z_{1} )_{1} - \frac{1}{2}h\left( {\Pi (z_{2} )_{2} + \Pi (z_{2} )_{1} } \right) = (r_{2} )_{{1 - \frac{1}{2}}} , $$

57 $$ \Pi \theta_{1} - \Pi \theta_{0} - \frac{1}{2}h\left( {\Pi (z_{3} )_{2} + \Pi (z_{3} )_{1} } \right) = (r_{3} )_{{1 - \frac{1}{2}}} , $$

58 $$ \begin{aligned} & (a_{1} )_{2} \Pi f_{2} + (a_{2} )_{2} \Pi f_{1} + (a_{3} )_{2} \Pi z_{12} + (a_{4} )_{2} \Pi z_{11} + (a_{5} )_{2} \Pi z_{22} + (a_{6} )_{2} \Pi z_{21} \\ & \quad + (a_{7} )_{2} \Pi \theta_{2} + (a_{8} )_{2} \Pi \theta_{1} + (a_{9} )_{2} \Pi (z_{3} )_{2} + (a_{10} )_{2} \Pi (z_{3} )_{1} = (r_{4} )_{{2 - \frac{1}{2}}} , \\ \end{aligned} $$

59 $$ \begin{aligned} & (b_{1} )_{2} \Pi f_{2} + (b_{2} )_{2} \Pi f_{1} + (b_{3} )_{2} \Pi z_{12} + (b_{4} )_{2} \Pi z_{11} + (b_{5} )_{2} \Pi z_{22} + (b_{6} )_{2} \Pi z_{21} \\ & \quad + (b_{7} )_{2} \Pi \theta_{2} + (b_{8} )_{2} \Pi \theta_{1} + (b_{9} )_{2} \Pi (z_{3} )_{2} + (b_{10} )_{2} \Pi (z_{3} )_{1} = (r_{5} )_{{2 - \frac{1}{2}}} . \\ \end{aligned} $$

In array arrangement,

60 $$ \begin{aligned} & \left[ {\begin{array}{*{20}l} 0 \hfill & 0 \hfill & { - 1} \hfill & 0 \hfill & 0 \hfill \\ 0 \hfill & 0 \hfill & 0 \hfill & { - h/2} \hfill & 0 \hfill \\ 0 \hfill & 0 \hfill & 0 \hfill & 0 \hfill & { - h/2} \hfill \\ 0 \hfill & 0 \hfill & {(a_{4} )_{2} } \hfill & {(a_{2} )_{2} } \hfill & {(a_{10} )_{2} } \hfill \\ 0 \hfill & 0 \hfill & {(b_{4} )_{2} } \hfill & {(b_{2} )_{2} } \hfill & {(b_{10} )_{2} } \hfill \\ \end{array} } \right]\left[ {\begin{array}{*{20}l} {\Pi (z_{2} )_{0} } \hfill \\ {\Pi (\theta )_{0} } \hfill \\ {\Pi (f)_{1} } \hfill \\ {\Pi (z_{2} )_{1} } \hfill \\ {\Pi (z_{3} )_{1} } \hfill \\ \end{array} } \right] \\ & \quad + \left[ {\begin{array}{*{20}l} { - h/2} \hfill & 0 \hfill & 1 \hfill & 0 \hfill & 0 \hfill \\ { - 1} \hfill & 0 \hfill & 0 \hfill & { - h/2} \hfill & 0 \hfill \\ 0 \hfill & { - 1} \hfill & 0 \hfill & 0 \hfill & { - h/2} \hfill \\ {(a_{6} )_{2} } \hfill & {(a_{8} )_{2} } \hfill & {(a_{3} )_{2} } \hfill & {(a_{1} )_{2} } \hfill & {(a_{9} )_{2} } \hfill \\ {(b_{6} )_{2} } \hfill & {(b_{8} )_{2} } \hfill & {(b_{3} )_{2} } \hfill & {(b_{1} )_{2} } \hfill & {(b_{9} )_{2} } \hfill \\ \end{array} } \right]\left[ {\begin{array}{*{20}l} {\Pi (z_{1} )_{1} } \hfill \\ {\Pi (\theta )_{1} } \hfill \\ {\Pi (f)_{2} } \hfill \\ {\Pi (z_{2} )_{2} } \hfill \\ {\Pi (z_{3} )_{2} } \hfill \\ \end{array} } \right] \\ & \quad + \left[ {\begin{array}{*{20}l} { - h/2} \hfill & 0 \hfill & 1 \hfill & 0 \hfill & 0 \hfill \\ 1 \hfill & 0 \hfill & 0 \hfill & { - h/2} \hfill & 0 \hfill \\ 0 \hfill & 1 \hfill & 0 \hfill & 0 \hfill & { - h/2} \hfill \\ {(a_{5} )_{2} } \hfill & {(a_{7} )_{2} } \hfill & 0 \hfill & 0 \hfill & 0 \hfill \\ {(b_{5} )_{2} } \hfill & {(b_{7} )_{2} } \hfill & 0 \hfill & 0 \hfill & 0 \hfill \\ \end{array} } \right]\left[ {\begin{array}{*{20}l} {\Pi (z_{1} )_{1} } \hfill \\ {\Pi (\theta )_{1} } \hfill \\ {\Pi (f)_{2} } \hfill \\ {\Pi (z_{2} )_{2} } \hfill \\ {\Pi (z_{3} )_{2} } \hfill \\ \end{array} } \right] = \left[ {\begin{array}{*{20}l} {(r_{1} )_{\frac{3}{2}} } \hfill \\ {(r_{2} )_{\frac{3}{2}} } \hfill \\ {(r_{3} )_{\frac{3}{2}} } \hfill \\ {(r_{4} )_{\frac{3}{2}} } \hfill \\ {(r_{5} )_{\frac{3}{2}} } \hfill \\ \end{array} } \right]. \\ \end{aligned} $$

That is

61 $$ \left[ {B_{2} } \right]\left[ {\Pi_{1} \left] + \right[A_{2} } \right]\left[ {\Pi_{2} \left] + \right[C_{2} } \right]\left[ {\Pi_{3} \left] = \right[r_{2} } \right]. $$

For $$j = J - 1;$$

62 $$ \Pi f_{J - 1} - \Pi f_{J - 2} - \frac{1}{2}h\left( {\Pi (z_{1} )_{J - 1} + \Pi z_{1J - 2} } \right) = (r_{1} )_{{J - 1 - \frac{1}{2}}} , $$

63 $$ \Pi (z_{1} )_{J - 1} - \Pi (z_{1} )_{J - 2} - \frac{1}{2}h\left( {\Pi (z_{2} )_{J - 1} + \Pi (z_{2} )_{J - 2} } \right) = (r_{2} )_{{J - 1 - \frac{1}{2}}} , $$

64 $$ \Pi \theta_{J - 1} - \Pi \theta_{J - 2} - \frac{1}{2}h\left( {\Pi (z_{3} )_{J - 1} + \Pi (z_{3} )_{J - 2} } \right) = (r_{3} )_{{J - 1 - \frac{1}{2}}} , $$

65 $$ \begin{aligned} & (a_{1} )_{J - 1} \Pi f_{J - 1} + (a_{2} )_{J - 1} \Pi f_{J - 2} + (a_{3} )_{J - 1} \Pi z_{1J - 1} + (a_{4} )_{J - 1} \Pi z_{1J - 2} \\ & \quad + (a_{5} )_{J - 1} \Pi z_{2j} + (a_{6} )_{J - 1} \Pi z_{2J - 2} + (a_{7} )_{J - 1} \Pi \theta_{J - 1} + (a_{8} )_{J - 1} \Pi \theta_{J - 2} \\ & \quad + (a_{9} )_{J - 1} \Pi (z_{3} )_{J - 1} + (a_{10} )_{J - 1} \Pi (z_{3} )_{J - 2} = (r_{4} )_{{J - 1 - \frac{1}{2}}} , \\ \end{aligned} $$

66 $$ \begin{aligned} & (b_{1} )_{J - 1} \Pi f_{J - 1} + (b_{2} )_{J - 1} \Pi f_{J - 2} + (b_{3} )_{J - 1} \Pi z_{1J - 1} + (b_{4} )_{J - 1} \Pi z_{1J - 2} \\ & \quad + (b_{5} )_{J - 1} \Pi z_{2J - 1} + (b_{6} )_{J - 1} \Pi z_{2J - 2} + (b_{7} )_{J - 1} \Pi \theta_{J - 1} + (b_{8} )_{J - 1} \Pi \theta_{J - 2} \\ & \quad + (b_{9} )_{J - 1} \Pi (z_{3} )_{J - 1} + (b_{10} )_{J - 1} \Pi (z_{3} )_{J - 2} = (r_{5} )_{{J - 1 - \frac{1}{2}}} . \\ \end{aligned} $$

In array arrangement,

67 $$ \begin{aligned} & \left[ {\begin{array}{*{20}l} 0 \hfill & 0 \hfill & { - 1} \hfill & 0 \hfill & 0 \hfill \\ 0 \hfill & 0 \hfill & 0 \hfill & { - h/2} \hfill & 0 \hfill \\ 0 \hfill & 0 \hfill & 0 \hfill & 0 \hfill & { - h/2} \hfill \\ 0 \hfill & 0 \hfill & {(a_{4} )_{J - 2} } \hfill & {(a_{2} )_{J - 2} } \hfill & {(a_{10} )_{J/2} } \hfill \\ 0 \hfill & 0 \hfill & {(b_{4} )_{J - 2} } \hfill & {(b_{2} )_{J - 2} } \hfill & {(b_{10} )_{J - 2} } \hfill \\ \end{array} } \right]\left[ {\begin{array}{*{20}l} {\Pi (z_{2} )_{J - 3} } \hfill \\ {\Pi (\theta )_{J - 3} } \hfill \\ {\Pi (f)_{J - 2} } \hfill \\ {\Pi (z_{2} )_{J - 2} } \hfill \\ {\Pi (z_{3} )_{J - 2} } \hfill \\ \end{array} } \right] \\ & \quad + \left[ {\begin{array}{*{20}l} { - h/2} \hfill & 0 \hfill & 1 \hfill & 0 \hfill & 0 \hfill \\ { - 1} \hfill & 0 \hfill & 0 \hfill & { - h/2} \hfill & 0 \hfill \\ 0 \hfill & { - 1} \hfill & 0 \hfill & 0 \hfill & { - h/2} \hfill \\ {(a_{6} )_{J - 2} } \hfill & {(a_{8} )_{J - 2} } \hfill & {(a_{3} )_{J - 2} } \hfill & {(a_{1} )_{J - 2} } \hfill & {(a_{9} )_{J - 2} } \hfill \\ {(b_{6} )_{J - 2} } \hfill & {(b_{8} )_{J - 2} } \hfill & {(b_{3} )_{J - 2} } \hfill & {(b_{1} )_{J - 2} } \hfill & {(b_{9} )_{J - 2} } \hfill \\ \end{array} } \right]\left[ {\begin{array}{*{20}l} {\Pi (z_{2} )_{J - 2} } \hfill \\ {\Pi (\theta )_{J - 2} } \hfill \\ {\Pi (f)_{J - 1} } \hfill \\ {\Pi (z_{2} )_{J - 1} } \hfill \\ {\Pi (z_{3} )_{J - 1} } \hfill \\ \end{array} } \right] \\ & \quad + \left[ {\begin{array}{*{20}l} { - h/2} \hfill & 0 \hfill & 0 \hfill & 0 \hfill & 0 \hfill \\ 1 \hfill & 0 \hfill & 0 \hfill & 0 \hfill & 0 \hfill \\ 0 \hfill & 1 \hfill & 0 \hfill & 0 \hfill & 0 \hfill \\ {(a_{5} )_{J - 2} } \hfill & {(a_{9} )_{J - 2} } \hfill & 0 \hfill & 0 \hfill & 0 \hfill \\ {(b_{5} )_{J - 2} } \hfill & {(b_{9} )_{J - 2} } \hfill & 0 \hfill & 0 \hfill & 0 \hfill \\ \end{array} } \right]\left[ {\begin{array}{*{20}l} {\Pi (z_{1} )_{J - 1} } \hfill \\ {\Pi (\theta )_{J - 1} } \hfill \\ {\Pi (f)_{J} } \hfill \\ {\Pi (z_{2} )_{J} } \hfill \\ {\Pi (z_{3} )_{J} } \hfill \\ \end{array} } \right] = \left[ {\begin{array}{*{20}l} {(r_{1} )_{{\left( {J - 1} \right) - \frac{1}{2}}} } \hfill \\ {(r_{2} )_{{\left( {J - 1} \right) - \frac{1}{2}}} } \hfill \\ {(r_{3} )_{{\left( {J - 1} \right) - \frac{1}{2}}} } \hfill \\ {(r_{4} )_{{\left( {J - 1} \right) - \frac{1}{2}}} } \hfill \\ {(r_{5} )_{{\left( {J - 1} \right) - \frac{1}{2}}} } \hfill \\ \end{array} } \right]. \\ \end{aligned} $$

That is

68 $$ \left[ {B_{J - 1} } \right]\left[ {\Pi_{J - 2} \left] + \right[A_{J - 1} } \right]\left[ {\Pi_{J - 1} \left] + \right[C_{J - 1} } \right]\left[ {\Pi_{J} \left] = \right[r_{J - 1} } \right]. $$

For $$j = J;$$

69 $$ \Pi f_{J} - \Pi f_{J - 1} - \frac{1}{2}h\left( {\Pi (z_{1} )_{J} + \Pi (z_{1} )_{J - 1} } \right) = (r_{1} )_{{J - \frac{1}{2}}} , $$

70 $$ \Pi (z_{1} )_{J} - \Pi (z_{1} )_{J - 1} - \frac{1}{2}h\left( {\Pi (z_{2} )_{J} + \Pi (z_{2} )_{J - 1} } \right) = (r_{2} )_{{J - \frac{1}{2}}} , $$

71 $$ \Pi \theta_{J} - \Pi \theta_{J - 1} - \frac{1}{2}h\left( {\Pi (z_{3} )_{J} + \Pi (z_{3} )_{J - 1} } \right) = (r_{3} )_{{J - \frac{1}{2}}} , $$

72 $$ \begin{aligned} & (a_{1} )_{J} \Pi f_{J} + (a_{2} )_{J} \Pi f_{J - 1} + (a_{3} )_{J} \Pi z_{1J} + (a_{4} )_{J} \Pi z_{1J - 1} + (a_{5} )_{J} \Pi z_{2J} + (a_{6} )_{J} \Pi z_{2J - 1} \\ & \quad + (a_{7} )_{J} \Pi \theta_{J} + (a_{8} )_{J} \Pi \theta_{J - 1} + (a_{9} )_{J} \Pi (z_{3} )_{J} + (a_{10} )_{J} \Pi (z_{3} )_{J - 1} = (r_{4} )_{{J - \frac{1}{2}}} , \\ \end{aligned} $$

73 $$ \begin{aligned} & (b_{1} )_{J} \Pi f_{J} + (b_{2} )_{J} \Pi f_{J - 1} + (b_{3} )_{J} \Pi z_{1J} + (b_{4} )_{J} \Pi z_{1J - 1} + (b_{5} )_{J} \Pi z_{2J} + (b_{6} )_{J} \Pi z_{2J - 1} \\ & \quad + (b_{7} )_{J} \Pi \theta_{J} + (b_{8} )_{J} \Pi \theta_{J - 1} + (b_{9} )_{J} \Pi (z_{3} )_{J} + (b_{10} )_{J} \Pi (z_{3} )_{J - 1} = (r_{5} )_{{J - \frac{1}{2}}} . \\ \end{aligned} $$

In matrix arrangement,

74 $$ \begin{aligned} & \left[ {\begin{array}{*{20}l} { - h/2} \hfill & 0 \hfill & 1 \hfill & 0 \hfill & 0 \hfill \\ { - 1} \hfill & 0 \hfill & 0 \hfill & { - h/2} \hfill & 0 \hfill \\ 0 \hfill & { - 1} \hfill & 0 \hfill & 0 \hfill & { - h/2} \hfill \\ {(a_{6} )_{1} } \hfill & {(a_{8} )_{1} } \hfill & {(a_{3} )_{1} } \hfill & {(a_{1} )_{1} } \hfill & {(a_{9} )_{1} } \hfill \\ {(b_{6} )_{1} } \hfill & {(b_{8} )_{1} } \hfill & {(b_{3} )_{1} } \hfill & {(b_{1} )_{1} } \hfill & {(b_{9} )_{1} } \hfill \\ \end{array} } \right]\left[ {\begin{array}{*{20}l} {\Pi (z_{2} )_{0} } \hfill \\ {\Pi (\theta )_{0} } \hfill \\ {\Pi (f)_{1} } \hfill \\ {\Pi (z_{2} )_{1} } \hfill \\ {\Pi (z_{3} )_{1} } \hfill \\ \end{array} } \right] \\ & \quad + \left[ {\begin{array}{*{20}l} { - h/2} \hfill & 0 \hfill & 1 \hfill & 0 \hfill & 0 \hfill \\ { - 1} \hfill & 0 \hfill & 0 \hfill & { - h/2} \hfill & 0 \hfill \\ 0 \hfill & { - 1} \hfill & 0 \hfill & 0 \hfill & { - h/2} \hfill \\ {(a_{6} )_{J - 2} } \hfill & {(a_{8} )_{J - 2} } \hfill & {(a_{3} )_{J - 2} } \hfill & {(a_{1} )_{J - 2} } \hfill & {(a_{9} )_{J - 2} } \hfill \\ {(b_{6} )_{J - 2} } \hfill & {(b_{8} )_{J - 2} } \hfill & {(b_{3} )_{J - 2} } \hfill & {(b_{1} )_{J - 2} } \hfill & {(b_{9} )_{J - 2} } \hfill \\ \end{array} } \right]\left[ {\begin{array}{*{20}l} {\Pi (z_{2} )_{J - 2} } \hfill \\ {\Pi (\theta )_{J - 2} } \hfill \\ {\Pi (f)_{J - 1} } \hfill \\ {\Pi (z_{2} )_{J - 1} } \hfill \\ {\Pi (z_{3} )_{J - 1} } \hfill \\ \end{array} } \right] = \left[ {\begin{array}{*{20}l} {(r_{1} )_{\frac{1}{2}} } \hfill \\ {(r_{2} )_{\frac{1}{2}} } \hfill \\ {(r_{3} )_{\frac{1}{2}} } \hfill \\ {(r_{4} )_{\frac{1}{2}} } \hfill \\ {(r_{5} )_{\frac{1}{2}} } \hfill \\ \end{array} } \right]. \\ \end{aligned} $$

That is

75 $$ \left[ {B_{J} } \right]\left[ {\Pi_{J - 1} \left] + \right[A_{J} } \right]\left[ {\Pi_{J} \left] = \right[r_{J} } \right]. $$

### Stage 5: bulk elimination scheme

Engaging expressions (48)–(73), the obtained tridiagonal-block matrix is as follows:

76 $$ R{\Pi } = p, $$

where

77 $$ R = \left[ {\begin{array}{*{20}l} {A_{1} } \hfill & {C_{1} } \hfill & {} \hfill & {} \hfill & {} \hfill & {} \hfill \\ {B_{2} } \hfill & {A_{2} } \hfill & {C_{2} } \hfill & {} \hfill & {} \hfill & {} \hfill \\ {} \hfill & \ddots \hfill & \ddots \hfill & \ddots \hfill & {} \hfill & {} \hfill \\ {} \hfill & {} \hfill & \ddots \hfill & \ddots \hfill & \ddots \hfill & {} \hfill \\ {} \hfill & {} \hfill & {} \hfill & {B_{J - 1} } \hfill & {A_{J - 1} } \hfill & {C_{J - 1} } \hfill \\ {} \hfill & {} \hfill & {} \hfill & {} \hfill & {B_{J} } \hfill & {A_{J} } \hfill \\ \end{array} } \right],\;\;\Pi = \left[ {\begin{array}{*{20}l} {\Pi_{1} } \hfill \\ {\Pi_{2} } \hfill \\ \vdots \hfill \\ {\Pi_{j - 1} } \hfill \\ {\Pi_{j} } \hfill \\ \end{array} } \right],\;\;p = \left[ {\begin{array}{*{20}l} {(r_{1} )_{{j - \frac{1}{2}}} } \hfill \\ {(r_{2} )_{{j - \frac{1}{2}}} } \hfill \\ \vdots \hfill \\ {(r_{J - 1} )_{{j - \frac{1}{2}}} } \hfill \\ {(r_{J} )_{{j - \frac{1}{2}}} } \hfill \\ \end{array} } \right]. $$

$$R$$ is shown as $$J \times J$$ tridiagonal-block matrix for all $$5 \times 5$$ bulk-size, meanwhile $$\Pi$$ together with $$p$$ are vector columns of order $$J \times 1$$. Solution of $$\Pi$$ is obtained by factorizing LU. Factorization can be done only if matrix R is non-singular. However,$$ p$$ vector is obtained when $$R\Pi = p$$ works on a vector, that is directed to tridiagonal block matrix R. Next, factorization of bulk tridiagonal matirx R is devided into upper as well as lower triangular matrices. In this way, $$R = LU$$ is written as $$LU\Pi = p$$. Assume that $$U\Pi = y$$ results in $$Ly = p$$. So y can be obtained.solution for $$\Pi$$ is obtained with putting solution of y again into $$U\Pi = y.$$ In triangular matrices, replacement is done for further work.

## Verification of code

Verification is done for acquired results with the help of comparison with available literature^96,97^. Comparison of consistencies available in studies is summarized in Table 5. However, highly accurate outcomes about the present analysis are obtained.

**Table 5:** Comparison regarding the values of $- \theta^{\prime}\left( 0 \right)$ with $P_{r}$, with fixed $A = 0$, $\phi = 0$, $\phi_{hnf} = 0$, $E_{c} = 0$, ${\Lambda } = 0$, $N_{r} = 0$, $S = 0$ and $B_{i} \to 0$.

$P_{r}$	Das et al.^96^	Jamshed et al.^97^	Present
72 × 10^−2^	0.79876122	0.79876180	0.79876180
1 × 10^0^	1.00000000	1.00000000	1.00000000
3 × 10^0^	1.92357431	1.92357420	1.92357420
7 × 10^0^	3.07314679	3.07314651	3.07314651
10 × 10^0^	3.72055436	3.72055429	3.72055429

## Second law of thermodynamics: entropy generation

Entropy formation for considered model is^98^:

78 $$ E_{G} = \frac{{k_{hnf} }}{{{\yen}_{\infty }^{2} }}\left\{ {\left( {\frac{{\partial {\yen}}}{\partial y}} \right)^{2} + \frac{16}{3}\frac{{\sigma^{*} {\yen}_{\infty }^{3} }}{{\kappa^{*} \nu_{f} (\rho C_{p} )_{f} }}\left( {\frac{{\partial {\yen}}}{\partial y}} \right)^{2} } \right\} + \frac{{\mu_{hnf} }}{{{\yen}_{\infty } }}\left( {\frac{{\partial G_{1} }}{\partial y}} \right)^{2} + \frac{{\mu_{hnf} G_{1}^{2} }}{{k{\yen}_{\infty } }}. $$

Entropy analysis has following dimension-less expression:

79 $$ N_{G} = \frac{{{\yen}_{\infty }^{2} b^{2} E_{G} }}{{k_{f} \left( {{\yen}_{w} - {\yen}_{\infty } } \right)^{2} }}. $$

By formula (15), the non-dimensional entropy formula is:

80 $$ N_{G} = R_{e} \left[ {\phi_{d} \left( {1 + N_{r} } \right)\theta ^{{\prime}{2}} + \frac{1}{{\phi_{a} }}\frac{{B_{r} }}{{\Omega }}\left( {f^{{\prime\prime}{2}} + Kf^{{\prime}{2}} } \right)} \right]. $$

Here $$R_{e} $$ is representing Reynolds number, Brinkmann number is $$B_{r} $$ and $$\Omega$$ symbolizes the dimensionless temperature gradient.

## Results and discussion

The features of two kinds of sodium alginate-based nanofluids e.g., Cu–SA and GO–Cu/SA are numerically scrutinized by employing the Keller-Box Technique. To authenticate our computational technique, we equate our simulated consequences with the analytical ones, for the limiting case of classical Newtonian flow in the present geometry. The upshots of emerging flow parameters are enumerated via different graphical and tabulated numerical findings.

**Figure 6:**
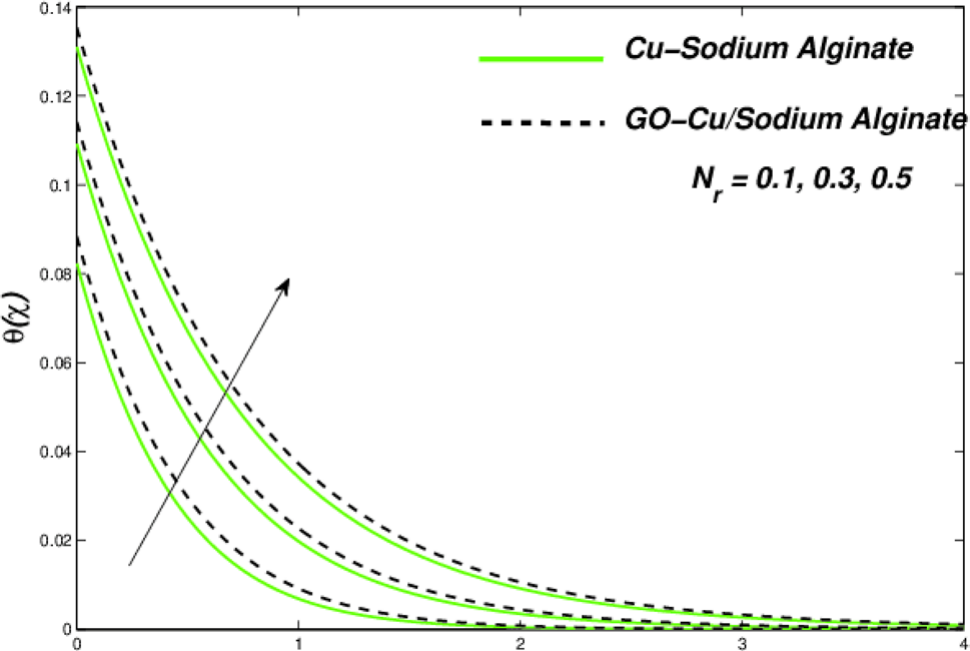
Variations in temperature regarding $N_{r}$.

**Figure 7:**
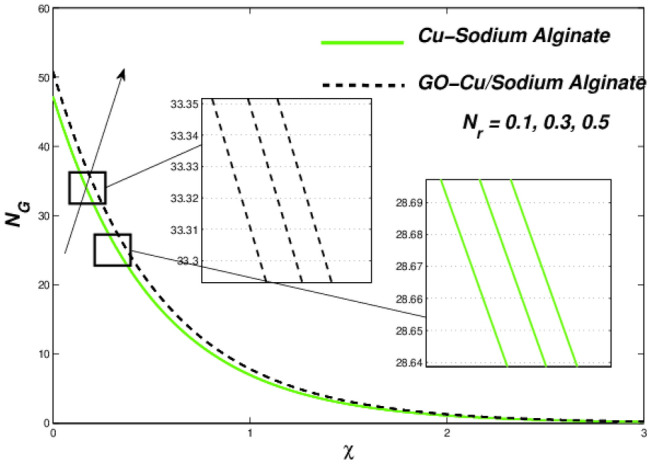
Variation in entropy regarding $N_{r}$.

**Figure 8:**
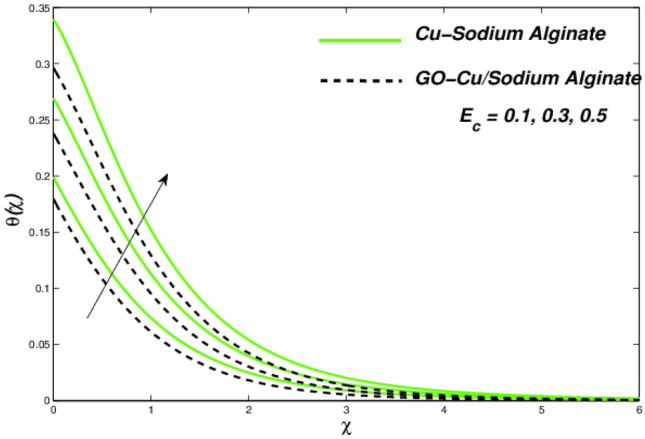
Temperature variations versus $E_{c}$.

**Figure 9:**
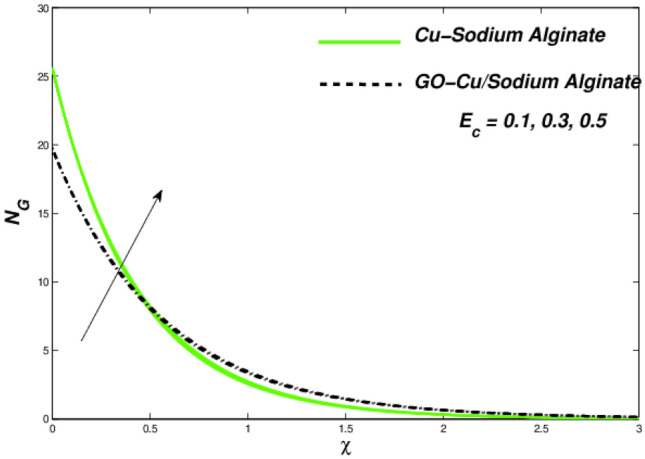
Entropy variations versus $E_{c}$.

**Figure 10:**
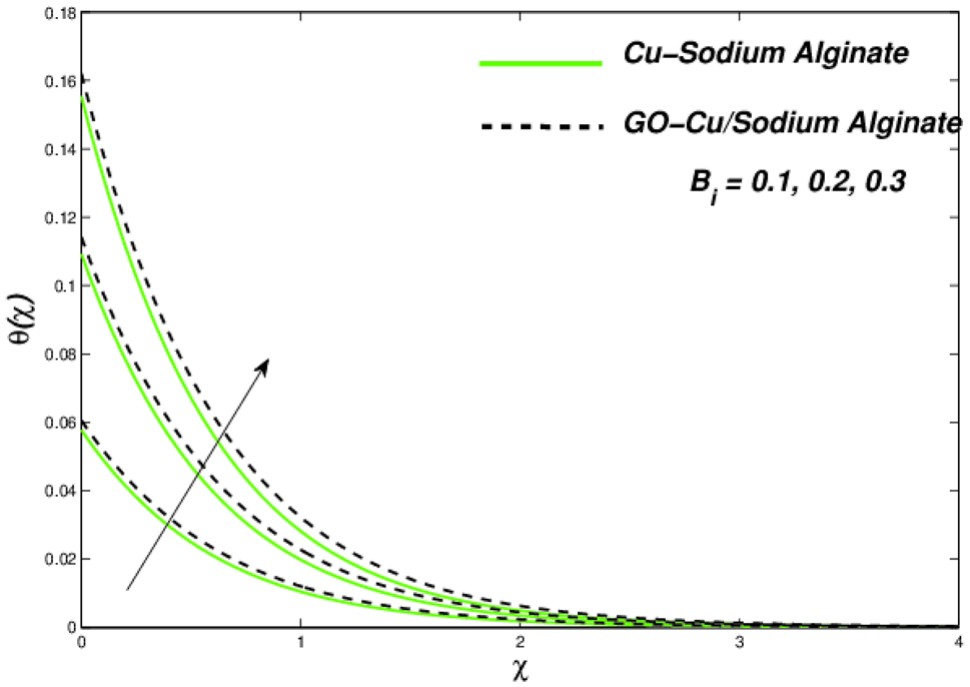
Temperature variations with $B_{i} .$

**Figure 11:**
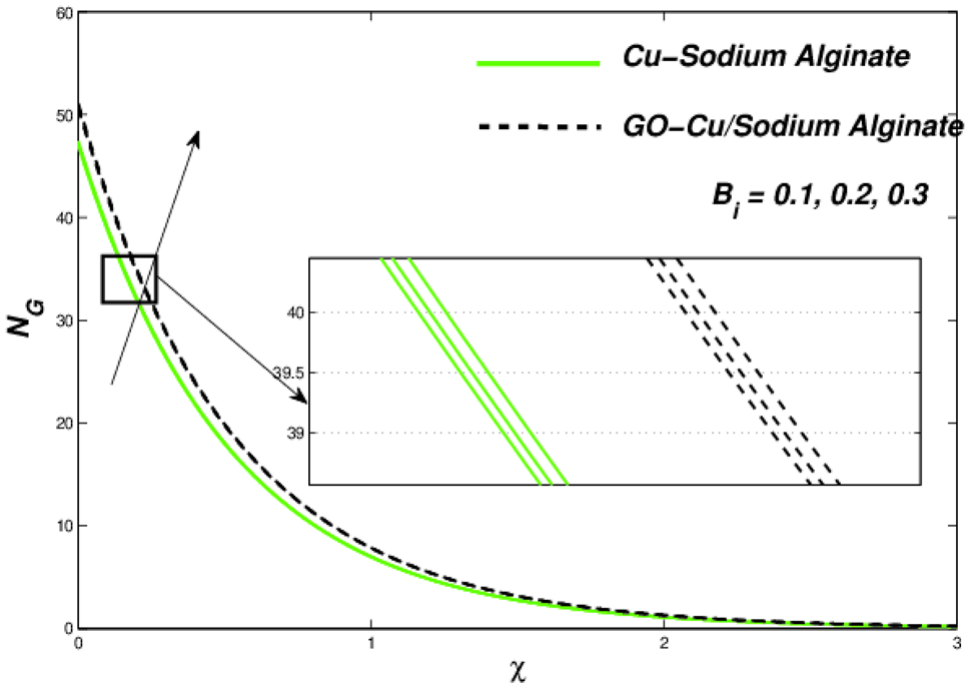
Entropy variations with variable $B_{i} .$

Figures 6 and 7 correspondingly evident sudden increase in entropy rate as well as thermal status with respect to induced temperature in domain of flow employing radiation process represented by factor of thermal radiation $$N_{r}$$. Furthermore, there is relatively little effect of radiation on fluctuations in entropy, which may be due to a strong influence on flow constraints Fig. 7. In this regard, Cu–SA nanofluid has a greater potential than GO–Cu/SA hybrid nanofluid. For hybrid GO–Cu/SA as well as Cu–SA fluid combo of nanofluids, variations in thermal as well as entropy formation regarding Eckert numbers ($$E_{c}$$) is represented in Figs. 8 and 9 respectively. Eckert number presents thermal fluctuation as well as entropy in both conditions. Internal friction of a fluid, when it is mixed with a temperature of the surface, the thermal condition regarding fluids improves. Effect on the temperature profile of Biot Number $$B_{i}$$ is portrayed in Fig. 10. The graph shows that the rising attitude of Biot Number $$B_{i}$$ assessed the temperature profiles. For small entities of $$B_{i}$$, thin thermal strips are related i.e., usually, there are uniform temperatures in the body (nano polymer surface). Generally, Biot number too greater than 1 indicates thermally thick situations whenever temperature non-uniformity is obtained. Graphical behviour of $$N_{G}$$ aginst progressive values of Biot number $$B_{i} $$ in Fig. 11 explores that it is insensitive (gradual increase) to vary at the surface as compared to away from it. i.e. less enhancement is observed near the stretching walls. Away from the surface, when deceleration in entropy generation is substituted with increasing of Biot number.

**Figure 12:**
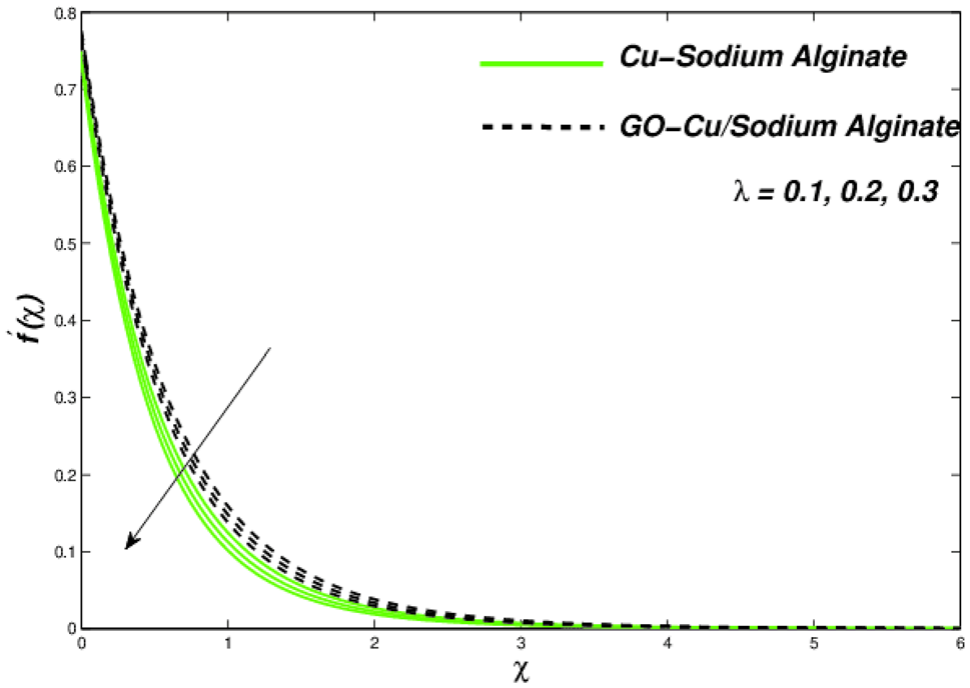
Changes in velocity subjected to $\lambda$.

**Figure 13:**
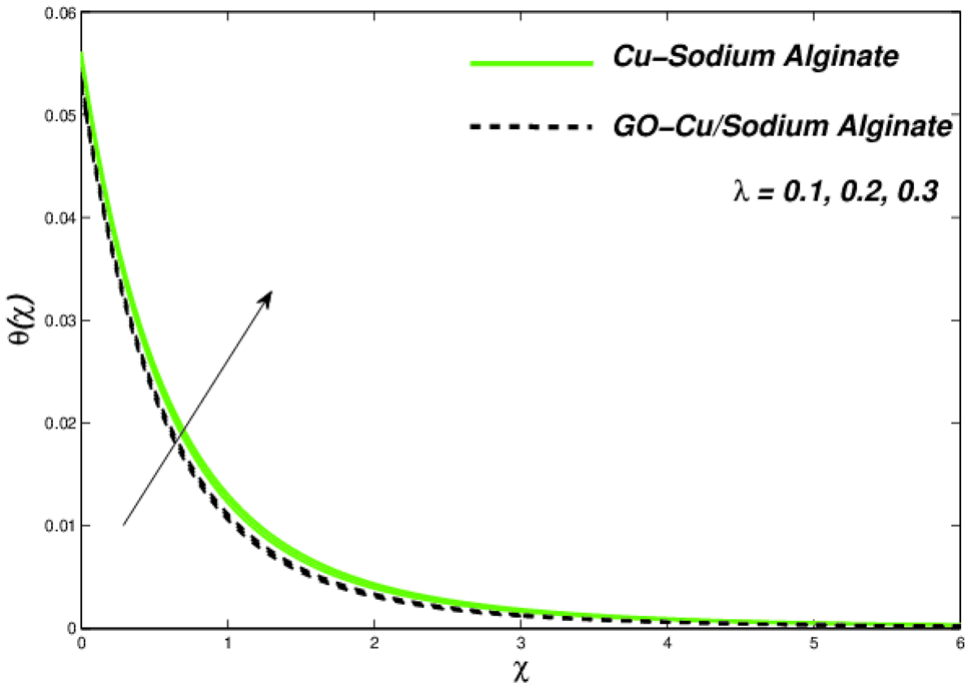
Changes in temperature subjected to $\lambda$.

**Figure 14:**
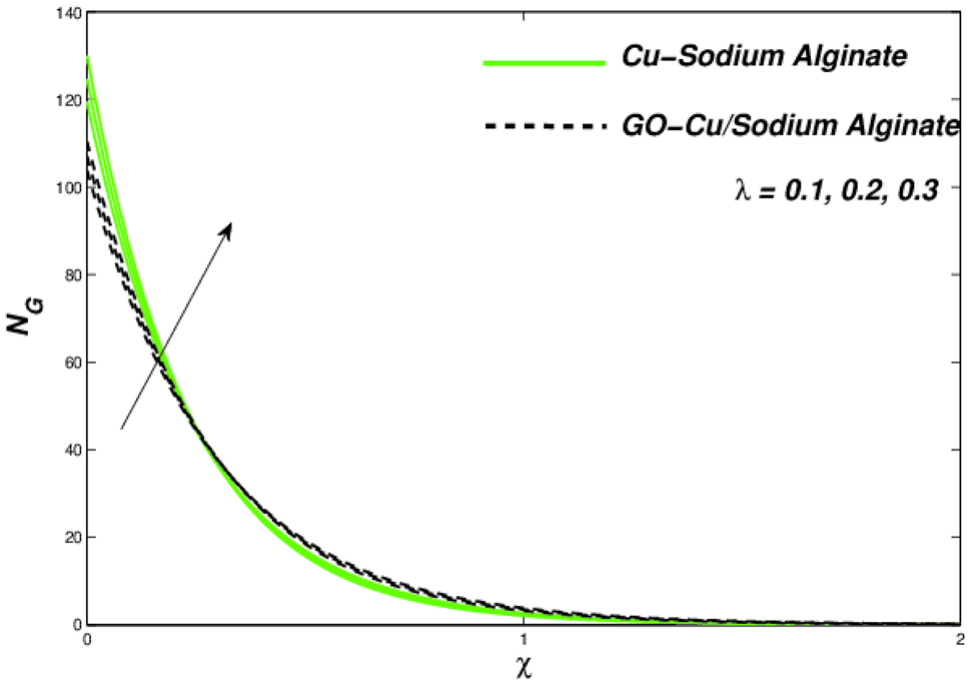
Entropy variation versus $\lambda$.

**Figure 15:**
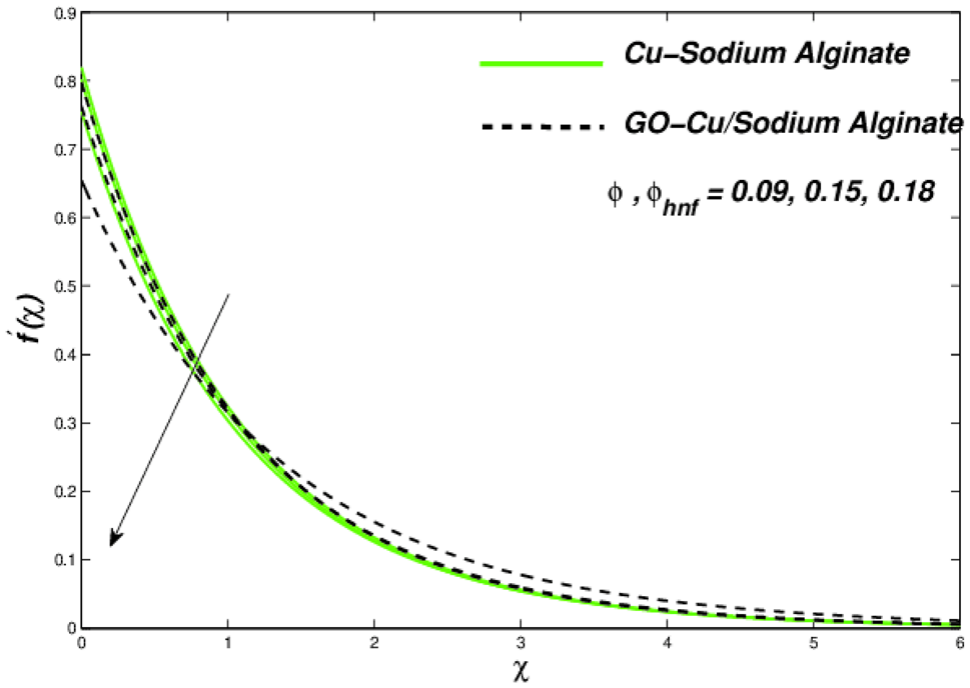
Velocity variation versus $\phi$ as well as $\phi_{hnf}$.

**Figure 16:**
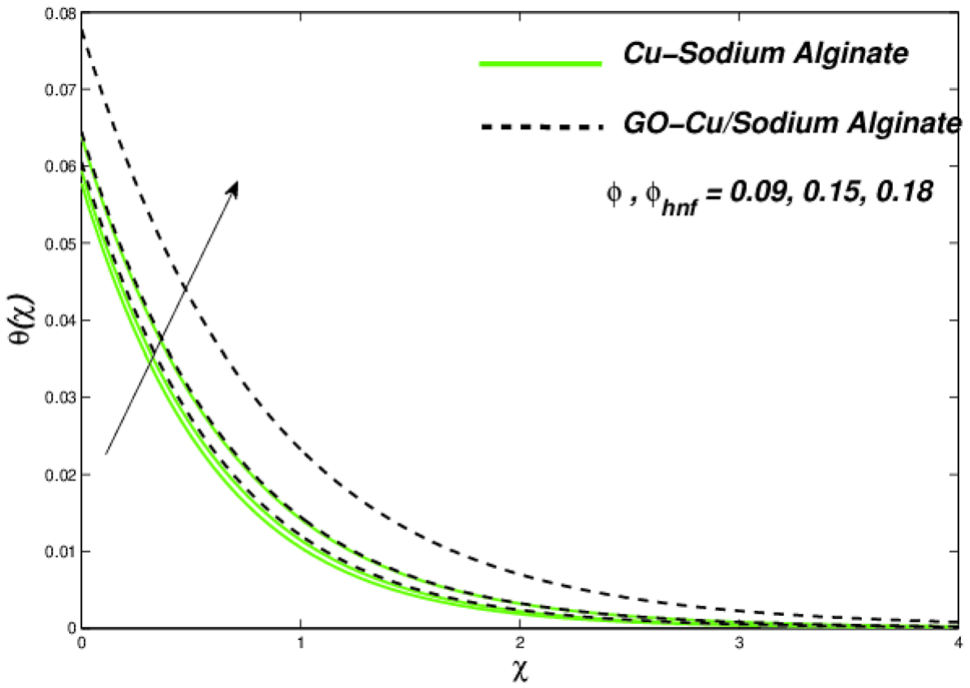
Temperature variations with respect to $\phi$ as well as $\phi_{hnf}$.

**Figure 17:**
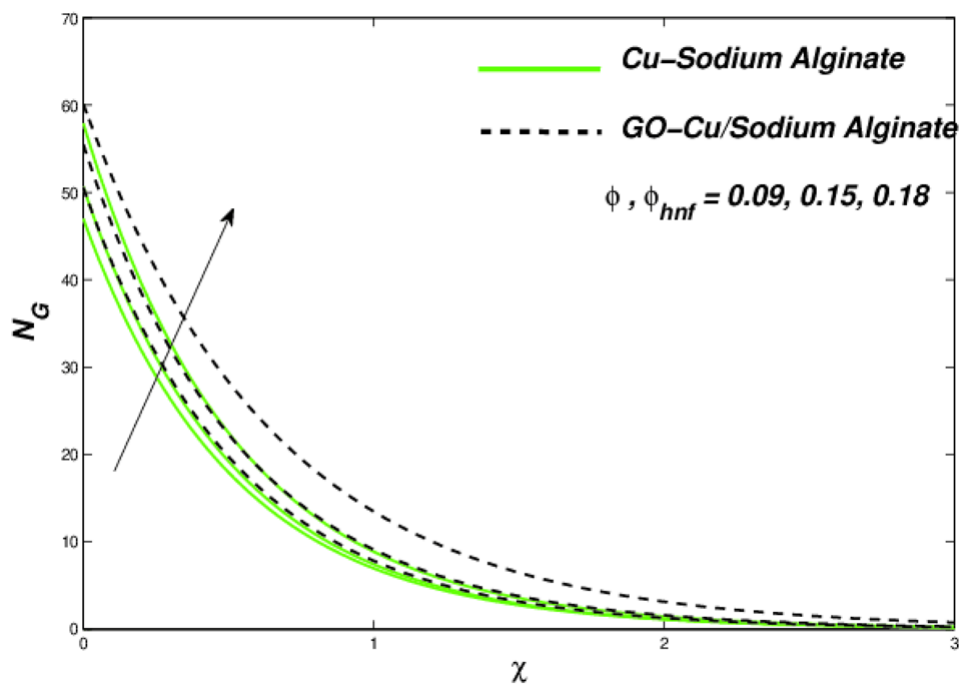
Entropy variations with respect to $\phi$ as well as $\phi_{hnf}$.

Figures 12, 13, and 14 clarify the viscidness-depended impacts over Williamson parameter $\lambda$. More than the flowing, thermal and entropy establishment of Cu–SA WNF together with hybridized form of GO–Cu/SA. Figure 12 shows flowing nature of together liquids for differing values of $\lambda$. In both cases, the material parameter tends to viscously repel the liquid flowing. Notwithstanding of HNF interruption, the hybridized liquid has greater velocity as related to its NF. Thermal position of Cu–SA WNF along with GO–Cu/SA WHNF for changing values of $\lambda$ portrayed in Fig. 13. Earlier revealed avoided flowing reveals an increase of fluid thermal state by delivering adequate ability to understand more heat form surface as the fluid flows over it deliberately. The viscosity-assisted positive entropy fluctuations for increasing values of $\lambda$ are shown in Fig. 14. Surprisingly, the conventional NF has more entropy production distant from -the wall. Nanoparticle volumetric fraction parameter $\phi$ effect toward velocity is shown in Fig. 15. As $\phi$ intensified, the speed of the fluid flow is lessened. This occurrence happens due to fluid viscosity rising with growing nanofluid concentration, friction escalations as well. The hybrid nanofluid has an advanced velocity than conventional nanofluid as $\phi$ augmented. It is found that the temperature of the system amplified along with $\phi$ in Fig. 16. It is value mentioning that fluid velocity is critical for heat transmission. The movement of the particles in the fluid slowed down will cause the heat to accumulate in the system. Hence, the temperature of the system doubles up Fig. 16. It is also expected that the system's entropy will amplify due to heat accumulation—this evidence is illustrated in Fig. 17. The results in Fig. 17 demonstrate that the nanoparticle volume fraction substantially influences the produced entropy. On the other hand, hybrid nanofluid generates less entropy than nanofluid, as seen in Fig. 17. This finding implies that hybrid nanofluids can better control the entropy system than nanofluids.

**Figure 18:**
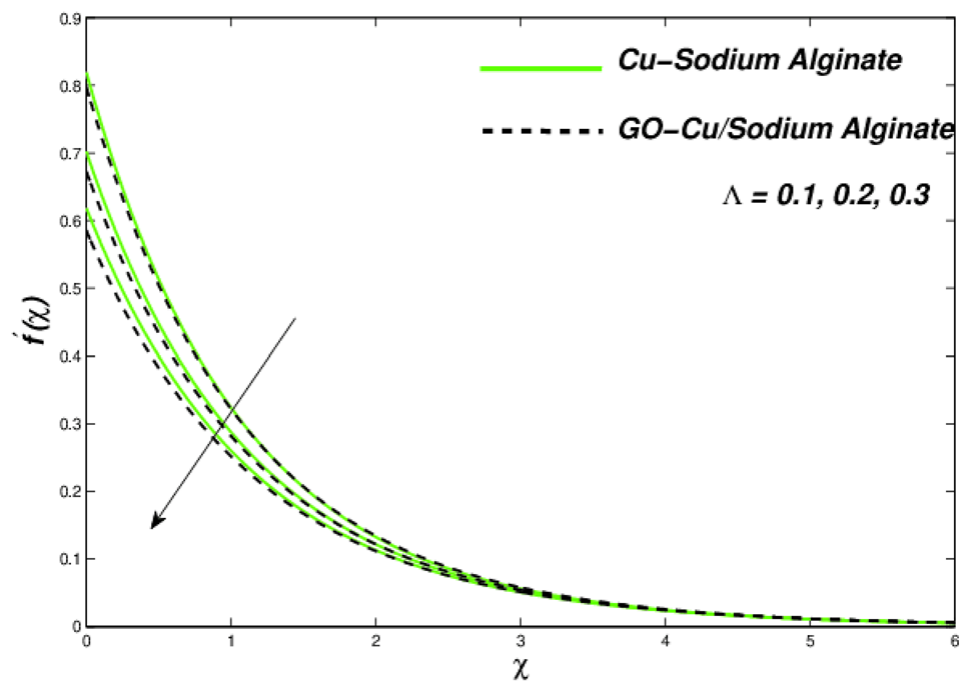
Velocity variations subject to ${\Lambda }$.

**Figure 19:**
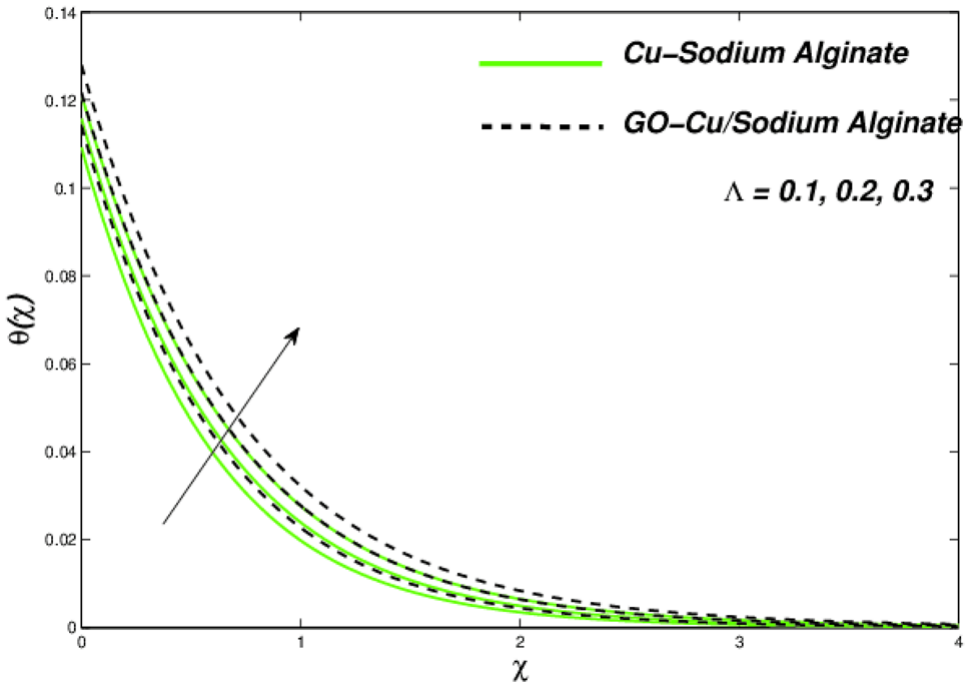
Temperature variations subject to ${\Lambda }$.

**Figure 20:**
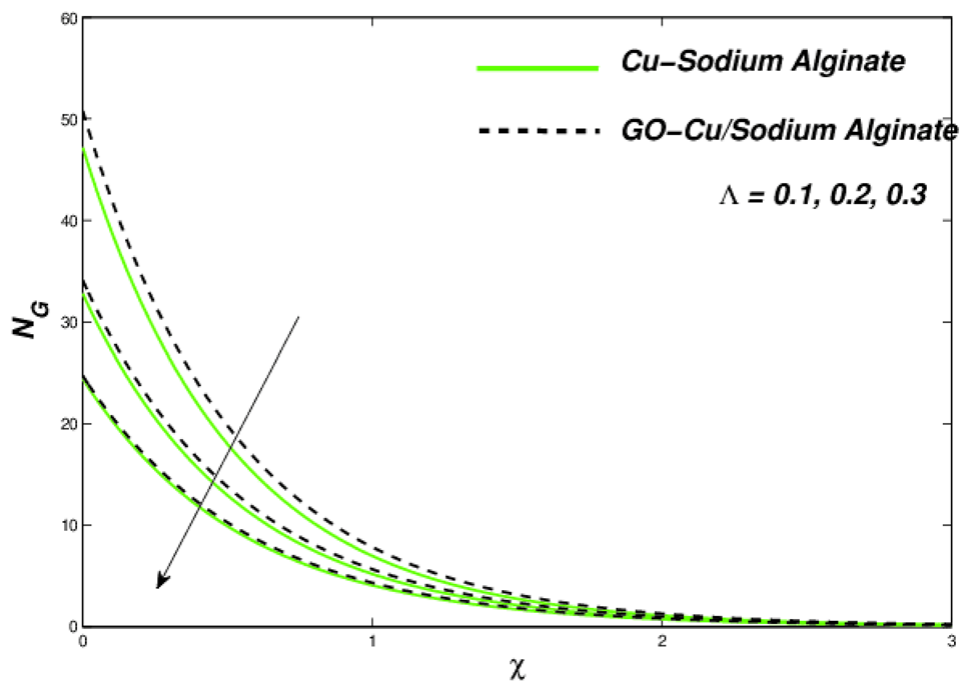
Entropy variations subject to ${\Lambda }$.

Figures 18, 19, and 20 presented the consequences due to improved slip conditions on flow nature, thermal aspects as well as entropy formations correspondingly. In Williamson fluid combinations, the flow situations mainly focused around the viscous behaviour. Along with it, the slip conditions becomes more crucial in all fluids facets such as velocity variations, thermal distributions as well as entropy generations. It can be noted that the viscous nature of the Sutterby fluid along with increased slip flow conditions, creates tougher situations for fluidity and makes it reducing further for single suspended nanofluid than that of hybrid suspended Williamson nanofluid. This flow hierarchy reflects in thermal distributions like Cu–SA nanofluid holds a greater thermal state as compared to GO–Cu/SA hybrid nanofluid (Fig. 19). A descending trend can be evident in entropy formation for larger amounts of slip parameters due to slip flow acting opposite to entropy formation across the domain (Fig. 20). The numerical observations of skin friction coefficients and wall temperature gradients against related flow parameters have been presented in Table 6.

**Table 6:** Values of $C_{f} Re_{x}^{1/2}$ and $Nu_{x} Re_{x}^{ - 1/2}$ for $P_{r} = 6.5$.

$\lambda$	$S$	$K$	$\phi$	$\phi_{\lambda }$	${\Lambda }$	$E_{c}$	$N_{r}$	$B_{i}$	$C_{f} Re_{x}^{\frac{1}{2}}$ Cu–SA	$C_{f} Re_{x}^{\frac{1}{2}}$ GO–Cu/SA	$N_{u} Re_{x}^{{\frac{ - 1}{2}}}$ Cu–SA	$N_{u} Re_{x}^{{\frac{ - 1}{2}}}$ GO–Cu/SA
0.1	0.1	0.1	0.18	0.09	0.3	0.2	0.3	0.2	2.1756	2.2793	1.1524	1.1857
0.2									1.1511	2.2485	1.1308	1.1662
0.3									1.1372	2.2226	1.1036	1.1465
	0.1								2.1756	2.2793	1.1524	1.1857
	0.2								2.1932	2.2918	1.1745	1.2089
	0.3								2.2142	2.3126	1.1880	1.2213
		0.1							2.1756	2.2793	1.1524	1.1857
		0.3							2.2048	2.3087	1.1703	1.2093
		0.4							2.2337	2.3295	1.1943	1.2276
			0.09						2.1424	-	1.1023	-
			0.15						2.1526	-	1.1241	-
			0.18						2.1756	-	1.1524	-
				0.0					-	2.1424	-	1.1023
				0.06					-	2.1860	-	1.1615
				0.09					-	2.2793	-	1.1857
					0.1				2.2356	2.3126	1.1017	1.2486
					0.2				2.2016	2.2938	1.1385	1.2194
					0.3				2.1756	2.2793	1.1524	1.1857
						0.1			2.1756	2.2793	1.1385	1.1419
						0.2			2.1756	2.2793	1.1524	1.1857
						0.3			2.1756	2.2793	1.1925	1.2236
							0.1		2.1756	2.2793	1.1252	1.1425
							0.3		2.1756	2.2793	1.1524	1.1857
							0.5		2.1756	2.2793	1.1827	1.2039
								0.1	2.1756	2.2793	1.1381	1.1558
								0.2	2.1756	2.2793	1.1524	1.1857
								0.3	2.1756	2.2793	1.1922	1.2160

## Final outcomes

In the current investigation, distinguished effects are investigated on hybrid nanofluid flow past a PTSC in solar-powered airplane wings. The present study is driven by the need to improve the phenomenon of solar energy, which will then be used in solar aviation for a variety of applications and a rise in aircraft perseverance. For this objective, Williamson hybrid nanofluid is considered. Tables along with plots are completely inspected and displayed for various parametrical effects: slated magnetic field effect, viscosity-based dissipation as well as the thermal liquid on PTSC together with a solar energy-based airplane. Coming up next are some significant outcomes from the current examination. Williamson hybrid nanofluid (GO–Cu/SA) is observed to be a better thermal conductor than ordinary Williamson nanofluid (Cu– SA). Velocity is diminished with a swelling impact of $\lambda$, $\phi$, and $\phi_{hnf}$. Higher concentration of nanoparticles resulted in an increasing heat transfer rate. System entropy is enhanced with increasing values of material factor, Reynolds number $R_{e}$, thermal radiation factor $N_{r}$, Brinkman number $B_{r}$ along with nanoparticle volumetric concentration parameter hnf while reduction is observed with the rise in parametric values of material as well as velocity slip.

### Future guidance

Outcomes of the current study can help in future improvements where the heat effect of the heating system may be assessed by taking into account different non-Newtonian hybrid nanofluids (i.e., Carreau, second-grade, Casson, Maxwell, micropolar nanofluids, etc.). Additionally, impacts of porosity, as well as viscosity that is dependent on temperature together with magneto-slip flow, can be represented by expanding technique.

## Data Availability

All data generated or analysed during this research investigation are included in this research article.
